# TNF-Signaling Modulates Neutrophil-Mediated Immunity at the Feto-Maternal Interface During LPS-Induced Intrauterine Inflammation

**DOI:** 10.3389/fimmu.2020.00558

**Published:** 2020-04-03

**Authors:** Pietro Presicce, Monica Cappelletti, Paranthaman Senthamaraikannan, Feiyang Ma, Marco Morselli, Courtney M. Jackson, Shibabrata Mukherjee, Lisa A. Miller, Matteo Pellegrini, Alan H. Jobe, Claire A. Chougnet, Suhas G. Kallapur

**Affiliations:** ^1^Divisions of Neonatology and Developmental Biology, David Geffen School of Medicine, University of California, Los Angeles, Los Angeles, CA, United States; ^2^Division of Neonatology/Pulmonary Biology, Cincinnati Children's Hospital Research Foundation, The University of Cincinnati College of Medicine, Cincinnati, OH, United States; ^3^Department of Molecular, Cell and Developmental Biology Medicine, University of California, Los Angeles, Los Angeles, CA, United States; ^4^Institute for Quantitative and Computational Biosciences–Collaboratory, University of California, Los Angeles, Los Angeles, CA, United States; ^5^Division of Immunobiology, Cincinnati Children's Hospital Research Foundation, The University of Cincinnati College of Medicine, Cincinnati, OH, United States; ^6^California National Primate Research Center, University of California, Davis, Davis, CA, United States; ^7^Department of Anatomy, Physiology, and Cell Biology, School of Veterinary Medicine, University of California, Davis, Davis, CA, United States

**Keywords:** chorioamnionitis, neutrophils, inflammation, innate immunity, reproductive immunology

## Abstract

Accumulation of activated neutrophils at the feto-maternal interface is a defining hallmark of intrauterine inflammation (IUI) that might trigger an excessive immune response during pregnancy. Mechanisms responsible of this massive neutrophil recruitment are poorly investigated. We have previously showed that intraamniotic injection of LPS in rhesus macaques induced a neutrophil predominant inflammatory response similar to that seen in human IUI. Here, we demonstrate that anti-TNF antibody (Adalimumab) inhibited ~80% of genes induced by LPS involved in inflammatory signaling and innate immunity in chorio-decidua neutrophils. Consistent with the gene expression data, TNF-blockade decreased LPS-induced neutrophil accumulation and activation at the feto-maternal interface. We also observed a reduction in IL-6 and other pro-inflammatory cytokines but not prostaglandins concentrations in the amniotic fluid. Moreover, TNF-blockade decreased mRNA expression of inflammatory cytokines in the chorio-decidua but not in the uterus, suggesting that inhibition of TNF-signaling decreased the inflammation in a tissue-specific manner within the uterine compartment. Taken together, our results demonstrate a predominant role for TNF-signaling in modulating the neutrophilic infiltration at the feto-maternal interface during IUI and suggest that blockade of TNF-signaling could be considered as a therapeutic approach for IUI, the major leading cause of preterm birth.

## Introduction

About 25–40% of preterm births (PTB) are associated with intrauterine infection (IUI) (1) and other studies suggest that inflammation is implicated in as many as 90% of very early PTB (2). Importantly, the causal link between IUI and PTB is well-established (3). Currently, the rate of prematurity remains stubbornly high at about 10% of all US births (4). Further, about 75% of perinatal mortality and 50% of the long-term childhood morbidity is directly attributable to prematurity (1). Apart from maternal morbidity, IUI causes fetal inflammation and increases the risk for fetal and newborn brain, gut, and lung injury in both clinical studies and in animal models [reviewed in (5)].

The histologic correlate of IUI is chorioamnionitis, defined as neutrophilic infiltration of the placenta, fetal membranes, and the amniotic fluid (6, 7). During IUI, neutrophils are the most abundant leukocyte, and are activated to secrete pro-inflammatory mediators (8–11). In contrast, small numbers of neutrophils are present in the chorio-decidua during normal gestation (12, 13). The roles of neutrophils in the chorio-decidua during normal pregnancy and IUI are not known. Thus, neutrophil function at the maternal-fetal interface during normal pregnancy and in pathologic inflammatory conditions needs further investigation to improve our understanding of normal and preterm labor.

Tumor necrosis factor α (TNFα) is implicated in IUI and PTB in clinical studies and animal experiments (14–16). Intra-amniotic (IA) injection of LPS in the rhesus macaque induced neutrophil infiltration of the chorio-decidua and increased neutrophil TNF expression in neutrophils (8, 10). Furthermore, IA infusion of TNFα alone induced intrauterine inflammation, neutrophil recruitment and preterm labor in rhesus macaques (17).

We therefore hypothesized that TNF-signaling was a major mediator of intrauterine inflammation. Our objective was to understand the role of TNF-signaling and particularly as it relates to neutrophils' recruitment and function during IUI. We tested this hypothesis in a rhesus macaque model of IUI induced by IA LPS, which was previously demonstrated to resemble human chorioamnionitis (10). TNF-signaling was inhibited by the anti-TNF antibody (Adalimumab—human IgG) used clinically to inhibit TNF in inflammatory diseases (18).

## Materials and Methods

### Animals

Normally cycling, adult female rhesus macaques (*Macaca mulatta*) (*n* = 56) were time mated. At ~130 d of gestation (~80% of term gestation), the pregnant rhesus received either a 1 ml saline solution (*n* = 26, two control animals received intramuscular instead of IA saline) or 1 mg LPS (Sigma-Aldrich, St. Louis, MO, *n* = 19) in 1 ml saline solution by ultrasound-guided intraamniotic (IA) injection. Tumor necrosis factor (TNF) signaling was inhibited in the amniotic and systemic compartments by the TNF blocker Adalimumab (Humira, AbbVie Inc. North Chicago, IL) given IA (40 mg) + maternal subcutaneous (s.c.) (40 mg) 1 and 3 h before LPS, respectively (*n* = 11) (Supplementary Figure 1). Fetuses were surgically delivered 16 h after LPS-exposure. This timing was determined to be the optimum time point based on our previous studies (10, 19). The multiparous macaques and their fetuses were similar in clinical variables (Supplementary Table 1). After delivery, fetuses were euthanized with pentobarbital, and fetal tissues were collected. There were no spontaneous deaths or preterm labor in the animals. The relatively large sample size was made possible by using tissues from animals used in a previous study (older samples) (10); Control (*n* = 16) and LPS exposed animals (*n* = 13) in addition to new animals: Controls (*n* = 10), LPS-exposed animals (*n* = 6), and Adalimumab-treated animals (*n* = 11) for the current study. It was not always possible to obtain all the tissues/fluids from each animal. The numbers of animals for each experiment are shown in the corresponding figure. All assays using older and newer samples were run at the same time. Comparison of data using older animals (tissues preserved longer in freezers) with the newer animals yielded similar results (not shown), and thus the combined data are shown. We confirmed bioavailability of specific TNF inhibitory activity in the amniotic fluid (AF) at 16 h (Supplementary Figure 2).

### Chorion-Amnion-Decidua Dissection

Extra-placental membranes were collected immediately after C-section and were dissected away from the placenta, as previously described (8, 10). After scraping decidua parietalis cells with the attached chorion, the amnion and rest of the chorion tissue were peeled away from each other with forceps. Chorio-decidua cells were washed, and digested with Dispase II (Life Technologies, Grand Island, NY) plus collagenase A (Roche, Indianapolis, IN) followed by DNase I (Roche) treatment, as previously described (8, 10). Cell suspensions were filtered, the red blood cells lysed and prepared for flow cytometry or FACS-sorting (10). Viability was >90% by trypan blue exclusion test.

### Flow Cytometry of Chorio-Decidua Cells

Monoclonal antibodies (mAbs) used for multiparameter flow cytometry (LSR Fortessa 2, BD Biosciences, San Diego, CA) are listed in the Supplementary Table 2. Gating strategy to identify the different leukocyte subpopulations was done as previously described (10). Cells were treated with 20 μg/mL human immunoglobulin G (IgG) to block Fc receptors, stained for surface markers for 30 min at 4°C in PBS, washed, and fixed in fixative stabilizing buffer (BD Bioscience). For detection of reactive oxygen species (ROS), 1 × 10^6^ chorio-decidua cells were loaded with 2.5 μM of Dihydrorhodamine123 (DHR, Molecular Probes, Eugene, OR) in DMSO or DMSO (as control). Cells were incubated at 37°C for 15 min. Following incubation, the samples were stained with a cocktail of mAbs (Supplementary Table 2) at room temperature in the dark for 30 min. Samples were acquired within 30 min after the staining and DHR MFI from neutrophils were compared to that of lymphocytes that lack this enzyme system activity (negative controls). All antibodies were titrated for optimal detection of positive populations and similar mean fluorescence intensity. At least 500,000 events were recorded for each sample. Doublets were excluded based on forward scatter properties, and dead cells were excluded using LIVE/DEAD Fixable Aqua dead cell stain (Life Technologies). Unstained and negative biological population were used to determine positive staining for each marker. Data were analyzed using FlowJo version 9.5.2 software (TreeStar Inc., Ashland, OR).

### Neutrophil Isolation

Maternal peripheral blood samples were collected 30 min before delivery from the same animals that the fetal membranes were collected. Blood neutrophils were isolated using human MACSxpress neutrophil isolation kit (Miltenyi Biotec, Auburn, CA). Neutrophil purity was >97%, as assessed by flow cytometry and Diff-Quick (Electron Microscopy Sciences, Hatfield, PA) staining of cytospin slides (Supplementary Figure 3). Chorio-decidua neutrophils cells were purified from chorio-decidua cell suspension by FACSAria Cell Sorter (BD Bioscience), using the same gating strategy as for immunophenotyping described previously (10). FACS-sorted chorio-decidua neutrophil purity was >97% (Supplementary Figure 4), as assessed by Diff-Quick staining. Total RNA was extracted from the purified neutrophils (2–4 × 10^6^) by adding TRIzol (ThermofisherScientific) and subsequently using Direct-Zol RNA MicroPrep kit (ZYMO Research, Irvine, CA) to efficiently extract small quantities of RNA.

### RNA Extraction and qPCR

Total RNA was extracted from fresh bead-sorted maternal blood neutrophils, FACS-sorted chorio-decidua neutrophils, snap-frozen chorioamnion-decidua, fetal lung, uterus, and amnion, after homogenizing in TRIzol (Invitrogen, Carlsbad, CA). RNA concentration and quality were measured by Nanodrop spectrophotometer (Thermo-Scientific). Reverse transcription of the RNA was performed using Verso cDNA synthesis kit (Thermo-Scientific). Quantitative RT-PCR was carried out in a StepOnePlus real-time PCR system (Life Technologies) following standard cycling conditions. Quantitative RT-PCR (qPCR) assays were performed with Rhesus-specific TaqMan gene expression primers (Life Technologies). A list of probes is provided in Supplementary Table 3. Eukaryotic 18S rRNA (Life Technologies) was the endogenous control for normalization of the target RNAs, and a sample from an IA saline injected rhesus animal was used to calibrate. The values were expressed relative to the average value of the control group.

### RNA Sequencing and Analysis

The integrity of purified total RNA from bead-isolated maternal blood neutrophils and FACS-sorted chorio-decidua neutrophils was assessed using HighSensitivity RNA ScreenTapes on the TapeStation 2200 (Agilent Technologies). Ten to fifty nanogram of starting material were used as input material for the NEBNext rRNA Depletion kit (cat# E6350). RNA libraries were then prepared using the NEBNext Ultra II RNA kit (cat# E7765). Quality control for each final library was performed using a D1000 ScreenTape (TapeStation 2200–Agilent Technologies) and quantified using a Qubit dsDNA BR Assay (Life Technologies). Diluted libraries were pooled and sequenced 50 single-end on a HiSeq3000 (Illumina). The reads were mapped with STAR 2.5.3a to the *Macaca mulatta* genome (Mmul 8.0.1). The counts for each gene were obtained by using quantMode GeneCounts in the STAR commands, and only counts for the featured genes were reserved. Differential expression analyses were carried out using DESeq2. The normalized counts were obtained from the DESeq2 analysis. Principal Component Analysis (PCA) were performed on the normalized counts using the open-source software AltAnalyze (http://www.altanalyze.org/). Volcano plots were made for the results from the differential expression analyses. Heatmaps were plotted on the log2 value of the normalized counts. Inference of biological processes were generated using Enrichr (20).

To determine maternal or fetal origin of chorio-decidua neutrophils, we used RNA-seq data from maternal blood neutrophils for maternal genotype and fetal lung for fetal genotype. We systemically scanned the entire RNA-seq data for sequences that were divergent between the mother and the fetus by aligning the bam files for fetal lung and maternal blood. We identified at least 5 such informative SNP divergence in genes with high expression in each mother-fetus pair (*n* = 3). The corresponding SNPs were compared between maternal neutrophils, fetal lung, and chorio-decidua neutrophils in the integrative genomics viewer (IGV). Final data were presented as number of reads and converted to frequency of nucleotides at each of the informative SNPs.

All original RNAseq data were deposited in the NCBI's Gene Expression Omnibus database (GEO accession: GSE145918).

### Cytokines and Prostaglandins ELISA

Cytokine/chemokine concentrations in amniotic fluid (AF), fetal, and maternal plasma were determined by Luminex using non-human primate multiplex kits (Millipore). Lipids were extracted from the AF using methanol and ELISA kits were used to measure Prostaglandins PGE_2_ (Oxford Biomedical Research, Oxford, MI) and PGF_2_α (Cayman Chemical, Ann Arbor, MI) concentrations.

### TNF Bio-Activity Experiments

Hek-Dual TNFα cells (Invivogen) derived from the human embryonic kidney 293 cell line by stable co-transfection of two NF-kB-inducible reporter constructs: the SEAP (secreted embryonic alkaline phosphatase) gene and the Lucia luciferase gene were used. Both reporter genes are under the control of the same NF-KB-inducible promoter. In this system, the cells specifically respond to TNF and not to other TLR/IL1 signals since they lack intracellular MyD88. Hek-Dual TNFα cells were stimulated over night with rTNFα (4 ng/ml, Peprotech) with or without the addition of amniotic fluid (AF) (dilution 1:10) obtained at delivery from Control (Ctrl) or Adalimumab injected animals. The presence or inhibition of bioactive TNFα was demonstrated by activation/inhibition of the AP-1/NF-KB pathway using QUANTI-Blue Solution (Invivogen) resulting in luciferase bio-luminiscence.

### Statistics

Prism version 5.0b software (GraphPad) was used to analyze data. Values were expressed as means ± SEM. Two-tailed Mann-Whitney *U*-tests and Unpaired *t*-test were used to determine differences between groups. Results were considered significant for *P* ≤ 0.05.

### Study Approval

All animal procedures were approved by the IACUC at UC Davis, California Primate Center.

## Results

### LPS-Exposure Induced a Neutrophil Predominant Immune Cell Infiltration of the Chorio-Decidua

**Figure 1:**
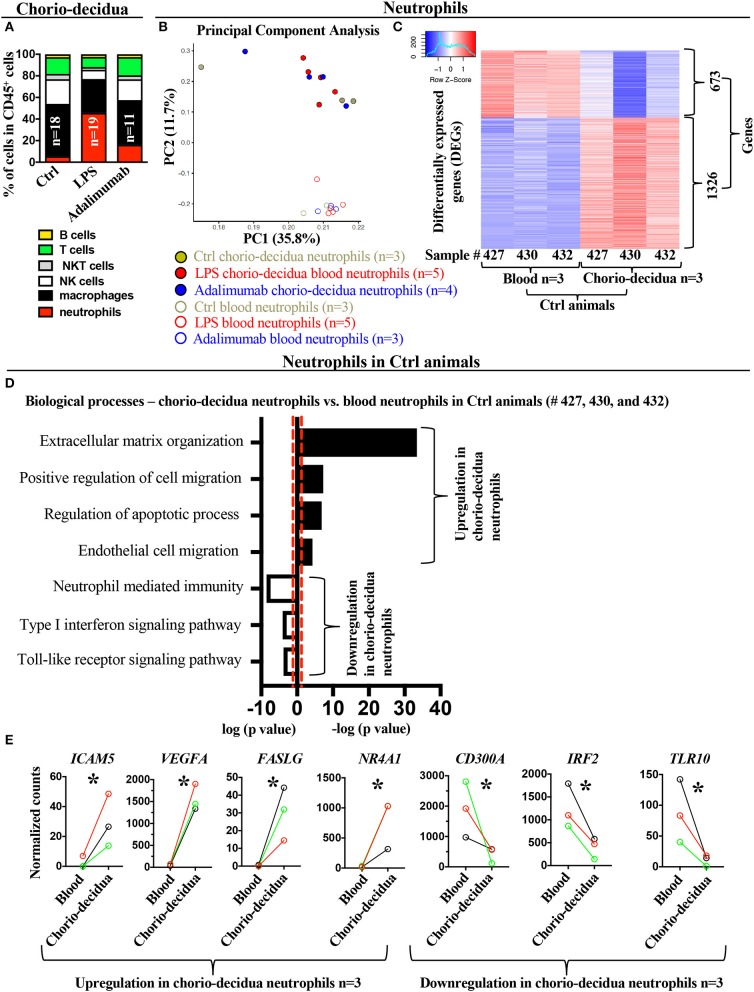
Changes in the global transcriptional landscape of chorio-decidua neutrophils compared to blood neutrophils. **(A)** Chorio-decidua cells were scraped, digested with protease/DNAase and single cell suspensions were used for multiparameter flow cytometry phenotyping. CD45^+^ subsets showed a neutrophil predominance after LPS (*n* = 19), while Adalimumab treatment (*n* = 11) partially restored the profile toward controls (*n* = 18). **(B)** Principal-component analysis (PCA) of RNA-seq profiling of neutrophils isolated from maternal blood using magnetic beads (open circles: Ctrl *n* = 3; LPS *n* = 5; Adalimumab *n* = 3) and from chorio-decidua by FACS (full circles: Ctrl *n* = 3; LPS *n* = 5; Adalimumab *n* = 4). The expression values were normalized across the entire datasets. **(C)** Heatmap of genes that are differentially expressed genes (DEGs, adjusted *p* < 0.05) between blood (*n* = 3) and chorio-decidua neutrophils (*n* = 3) in control animals only. **(D)** Biological processes of differentially expressed genes shown in **(C)**, as determined by Enrichr. Black bars represent upregulated biological processes and open bars represent downregulated biological processes in chorio-decidua neutrophils vs. blood neutrophils from control animals only. **(E)** Normalized counts of gene expression value of representative genes associated with the biological processes depicted in **(D)**. The lines show normalized counts of gene-expression trend plot from the same control animal (black line= sample #427, red line = sample #432, green line = sample #430; *n* = 3). ^*^p < 0.05 (Unpaired *t*-test).

During IUI, the chorio-decidua is the tissue with the highest infiltration of leukocytes at the feto-maternal interface (8, 10). Chorio-decidua immune cell profiling revealed that macrophage and NK cells were the most abundant leukocytes in controls, while neutrophils were the most abundant leukocytes in the chorio-decidua upon LPS-exposure (Figure 1A, Supplementary Figure 5). Adalimumab partially restored the chorio-decidua immune cell profile similar to controls (Figure 1A, Supplementary Figure 5). Besides neutrophils, LPS increased B cell counts and Adalimumab treatment reversed the B cell increases (Supplementary Figure 5).

### Blood vs. Chorio-Decidua Neutrophil Transcriptomes Differ Significantly

To determine the gene expression profile of the infiltrating neutrophils after LPS-exposure, we analyzed comparative gene expression profiles of blood and chorio-decidua neutrophils in controls and experimental animals. We first compared transcriptomic profiles from purified maternal blood neutrophils and FACS-sorted chorio-decidua neutrophils. Unbiased principal component analysis (PCA) of global gene-expression profiles demonstrated distinct clustering of blood vs. chorio-decidua neutrophils independent of prenatal exposures (Figure 1B), indicating that tissue of origin rather than treatments was a more important determinant of neutrophil gene-expression. In our model of localized intrauterine inflammation, maternal blood neutrophil gene expression profile did not differ significantly after IA LPS-exposure (Supplementary Figure 6) (also note similar PCA distribution between control and experimental blood neutrophil samples indicated in open circles in Figure 1B). Therefore, to better define gene signatures of chorio-decidua neutrophils, we restricted comparisons of chorio-decidua vs. blood neutrophils to control animals. Heat-maps of differentially expressed genes show distinct expression profiles in blood vs. chorio-decidua neutrophils (Figure 1C). Importantly, the replicates within each group were similar, demonstrating consistency of the findings. Functional annotations of differentially expressed genes predicted upregulation of the extracellular matrix organization, positive regulation of cell migration, regulation of apoptotic processes, and endothelial cell migration in the chorio-decidua compared to blood neutrophils (representative genes *ICAM5, VEGFA, FASLG, NR4A1*) (Figures 1D,E, Supplementary Figure 7A showing expanded list of representative genes for different pathways). Pathways predicted to be down-regulated in chorio-decidua were neutrophil-mediated immunity, type-I interferon signaling pathway, and toll-like receptor signaling (representative genes *CD300A, IRF2, TLR10*) (Figures 1D,E, Supplementary Figure 7B showing additional representative genes for different pathways). Notably, the pair-wise comparison of tissue vs. blood neutrophil was done using the same control animal lending additional validity to the analyses.

### Distinct Transcriptional Profiles in Chorio-Decidua Neutrophils Upon LPS-Exposure and TNF-Blockade

**Figure 2:**
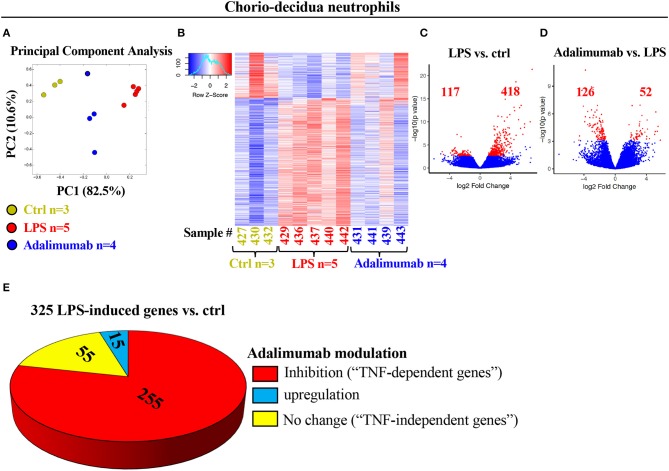
Profound changes in the global transcriptional landscape of chorio-decidua neutrophils upon different treatments. **(A)** Principal-component analysis of RNA-seq data from FACS-sorted chorio-decidua neutrophils of control (Ctrl) (green dots; *n* = 3), LPS (red dots; *n* = 5), and Adalimumab animals (blue dots; *n* = 4) showing distinct clustering based on exposures. **(B)** Heatmap of genes that are differentially expressed (DEGs) between chorio-decidua neutrophils upon different treatments showing minimal inter-animal variability within each group (Ctrl *n* = 3; LPS *n* = 5; Adalimumab *n* = 4). **(C,D)** Volcano plots displaying differentially expressed genes. Red dots and numbers indicate genes with an FDR-adjusted value <0.05. **(E)** Pie chart shows 325 genes that are induced by LPS (≥4-fold change vs. ctrl). Of those 325 genes, 225 genes are downregulated by Adalimumab (↓ ≥1.5-fold change vs. LPS, “TNF-dependent genes”), 55 do not change their expression upon Adalimumab treatment (↓1.5 < x < ↑1.5-fold change vs. LPS, “TNF-independent genes”), and 15 genes are upregulated by Adalimumab (≥↑1.5-fold change vs. LPS).

In contrast to blood neutrophils, PCA of global gene-expression profiles of FACS-sorted chorio-decidua neutrophils segregated according to prenatal exposures (Figure 2A). Heat-maps of differentially expressed genes were similar within each group and demonstrated distinct expression profiles in control vs. LPS-exposed neutrophils with an intermediate profile after TNF-blockade (Figure 2B). Comparison of LPS-exposed vs. control chorio-decidua neutrophils revealed 535 differentially expressed genes (418 upregulated, 117 downregulated), while 178 genes were differentially expressed in LPS vs. Adalimumab groups (Figures 2C,D). For further insight into the mechanisms of neutrophil activation during IUI, we focused on genes robustly induced after LPS (Base Mean >10 and fold change ≥4). Compared to control neutrophils, LPS-exposure upregulated a total of 325 transcripts (Figure 2E). Of these 325 LPS-induced genes, 255 genes were inhibited by Adalimumab (designated as “TNF-dependent genes”) (Base Mean > 10; fold decrease ≥ 1.5 in Adalimumab vs. LPS), 55 genes did not change their expression upon Adalimumab treatment (Base Mean > 10; fold change ↓1.5 < x < ↑1.5, “TNF-independent genes”) (Figure 2E), and only 15 genes were further up-regulated by Adalimumab (Figure 2E, Supplementary Table 4).

**Figure 3:**
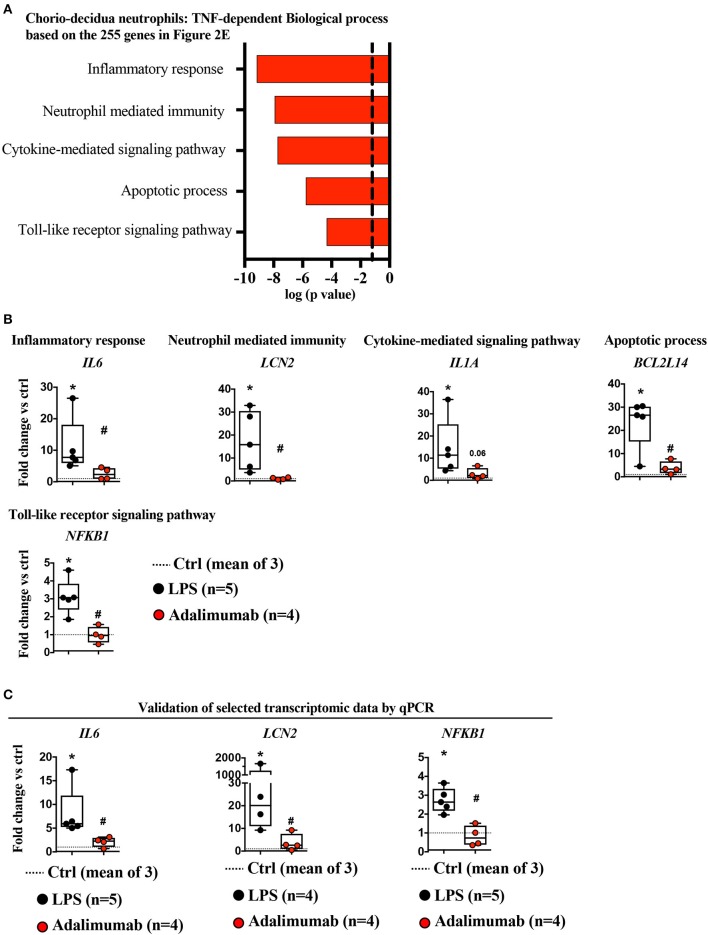
TNF-signaling inhibition decreased neutrophil mediated immunity. **(A)** Biological processes of LPS-induced genes (≥4-fold vs. controls) that were significantly down-regulated by Adalimumab (≥1.5-fold decrease vs. LPS) designated as “TNF-dependent genes,” determined using Enrichr. Dotted line represents gene-expression values representing the mean of three control animals. **(B)** Representative genes representing biological processes inhibited by Adalimumab shown in **(A)** (dotted-line represents the mean of 3 Ctrl; LPS *n* = 5; Adalimumab *n* = 4). **(C)** Validation of selected transcript expression by qPCR analysis. mRNAs were isolated from FACS-sorted chorio-decidua neutrophils from Ctrl (dotted-line represents the mean of 3 Ctrl), LPS- (*n* = 4–5), and Adalimumab-treated animals (*n* = 4). qPCR was performed using rhesus-specific Taqman probes. The values were first internally normalized to the endogenous 18S RNA. For each gene, the box plot (median and 5–95th percentile and outliers) shows fold-change of gene expression in chorio-decidua neutrophils normalized to control chorio-decidua neutrophils (dotted-line represents the mean of 3 ctrl). ^*^p < 0.05 vs. ctrl; #*p* < 0.05 vs. LPS (Mann–Whitney *U*-test).

Functional annotations of LPS-upregulated TNF-dependent genes predicted alterations in the following biological processes (*representative gene*): inflammatory response (*IL6*), neutrophil-mediated immunity (*LCN2*), cytokine-mediated signaling pathway (*IL1A*), apoptotic processes (*BCL2L14*), and toll-like receptor signaling (*NFkB1*) (Figures 3A,B, Supplementary Figure 8A, Supplementary Table 5). These findings were further validated by quantitative PCR (qPCR) analysis of a few selected key genes (e.g., *IL6, NFKB1, LCN2:* Figure 3C, and *TNF:* Supplementary Figure 8B–due to paucity of FACS-sorted neutrophil RNA, more extensive validation could not be performed). On the other hand, functional annotations of LPS-upregulated TNF-independent genes predicted changes in a number of biological processes including: negative regulation of signal transduction (*DKK1, SOCS3, CISH*), regulation of extracellular matrix organization (*DDR2*), positive regulation of cell migration (*PDPN2, SYDE1*), linoleic acid metabolic process (*FADS1, FADS2*) (Supplementary Figures 9A,B).

### Adalimumab Blocks Neutrophil Activation in Chorio-Decidua

**Figure 4:**
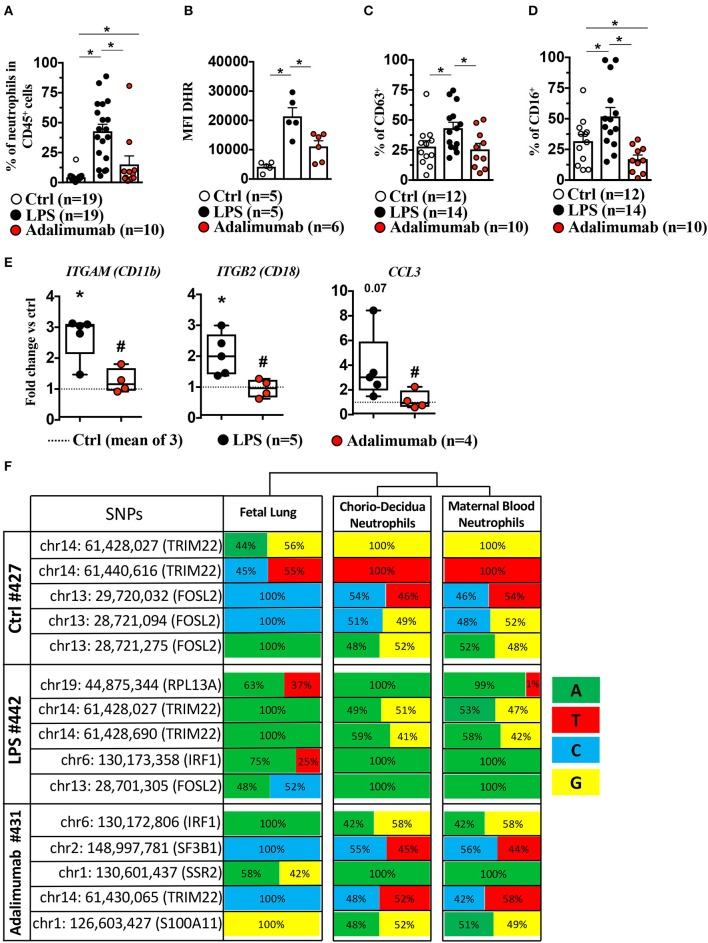
Adalimumab decreased LPS-induced chorio-decidua neutrophil accumulation and activation. Chorio-decidua cells were scraped and digested with protease/DNAase, and single cell suspensions were used for multiparameter flow cytometry. Neutrophils were defined as CD45^+^CD3^−^CD14^low^HLADR-CD88^+^ CD56^−^ cells. Inhibition of TNF-signaling by Adalimumab decreased significantly neutrophil frequency (Ctrl *n* = 19; LPS *n* = 19; Adalimumab *n* = 10) **(A)**, neutrophil reactive oxygen species production measured by Dihydro-rhodamine (DHR) fluorescence (Ctrl *n* = 5; LPS *n* = 5; Adalimumab *n* = 6) **(B)**, CD63 expression, a marker of degranulation of azurophilic granules (Ctrl *n* = 12; LPS *n* = 14; Adalimumab *n* = 10) **(C)**, and expression of FCγIII receptor CD16 (Ctrl *n* = 12; LPS *n* = 14; Adalimumab *n* = 10) **(D)**. Data are mean ± SEM, ^*^p < 0.05 between comparators by Mann-Whitney *U*-test. **(E)** Adalimumab decreased significantly the expression of genes associated with neutrophil chemotaxis. The box plot shows fold-change of gene expression in chorio-decidua neutrophils normalized to control chorio-decidua neutrophils (dotted-line represents the mean of 3 Ctrl; LPS *n* = 5; Adalimumab *n* = 4). ^*^p < 0.05 vs. ctrl; #*p* < 0.05 vs. LPS (Mann–Whitney *U*-test). **(F)** Frequency of nucleotides in SNPs in the fetal lung (representing fetal genome), chorio-decidua neutrophils, and maternal blood neutrophils (representing maternal genome) at five different informative loci in each mother-infant pair (total *n* = 3). Note similar frequency distribution of nucleotides at each of the SNPs in maternal blood neutrophils and chorio-decidua neutrophils and divergence compared to the fetal lung. For each SNP the chromosomal location and the encoded gene is shown. The frequency of nucleotides was computed at each SNP based on the number of reads shown in Supplementary Table 6.

We further validated genomic predictions of TNF-blockade of LPS-induced chorio-decidua neutrophil inflammatory pathways with biological measurements. LPS-exposure significantly increased the frequency of neutrophils (Figure 4A), the production of reactive oxygen species (ROS) in neutrophil as demonstrated by Dihydrorhodamine 123 (DHR) uptake (Figure 4B, Supplementary Figure 10A), and the expression of CD63, a marker for release of azurophilic granules (21)—(Figure 4C, Supplementary Figure 10B), and CD16 (FCRγIII) expression (22) (Figure 4D, Supplementary Figure 10C). Adalimumab efficiently reduced LPS-induced increases in frequency, ROS production, CD63/CD16 expression in chorio-decidua neutrophils, suggesting that TNF-signaling regulates the activation of neutrophils in the setting of IUI. In line with these results, Adalimumab also significantly reduced the expression of *ITGAM* (CD11b), *ITGB2* (CD18), and *CCL3*, suggesting that neutrophil chemotaxis is also regulated by TNF-signaling (Figure 4E). This finding is consistent with a previous study demonstrating that TNFα induced neutrophil migration through upregulation of integrin CD11b/CD18 and CCL3 (23).

To gain further insights into maternal vs. fetal origin of chorio-decidua neutrophils responding to LPS and TNF-signaling, we compared genotypes of the informative SNPs in three different mother-infant pairs (one each from the three groups). The nucleotide frequencies in each of the informative SNPs in all of the animals were almost identical in the maternal blood neutrophils and chorio-decidua neutrophils (Figure 4F and the corresponding reads shown in Supplementary Table 6). In comparison, fetal lung genotype showed a divergence in the nucleotide frequency indicating that the chorio-decidua neutrophils were almost entirely of maternal origin (Figure 4F and the corresponding reads shown in Supplementary Table 6).

### Adalimumab Blocks Inflammation in Chorioamnion-Decidua

**Figure 5:**
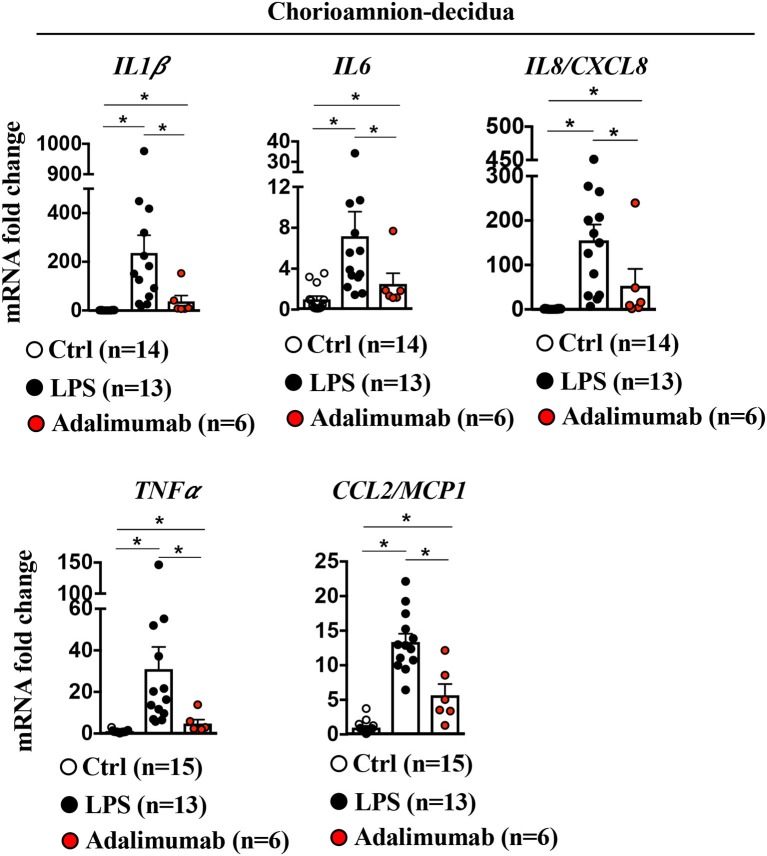
Adalimumab decreased inflammation in the chorioamnion-decidua tissue. Extraplacental chorion-amnion-decidua parietalis tissue (fetal membranes) was used. Expression of cytokine mRNAs by qPCR (Taqman probes) in the fetal membranes showed increased cytokines/chemokines after LPS, which was decreased after Adalimumab. Average mRNA values are fold increases over the average value for control after internally normalizing to the housekeeping 18S RNA (Ctrl *n* = 14–15; LPS *n* = 13; Adalimumab *n* = 6). Data are mean ± SEM, ^*^p < 0.05 vs. controls (Mann–Whitney *U*-test).

To understand if TNF-blockade of chorio-decidua neutrophil accumulation and activation was also associated with generalized anti-inflammatory effects, inflammatory mediators were assessed in multiple maternal-fetal compartments. LPS increased mRNAs for *IL1*β (200-fold), *IL6* (6-fold), *IL8* (150-fold), *TNF*α (30-fold), and *CCL2* (12-fold) in the chorioamnion-decidua tissue (Figure 5). Adalimumab broadly decreased LPS-induced increases in inflammatory mediators in the chorioamnion-decidua (Figure 5).

### Amnion Participation in IUI

**Figure 6:**
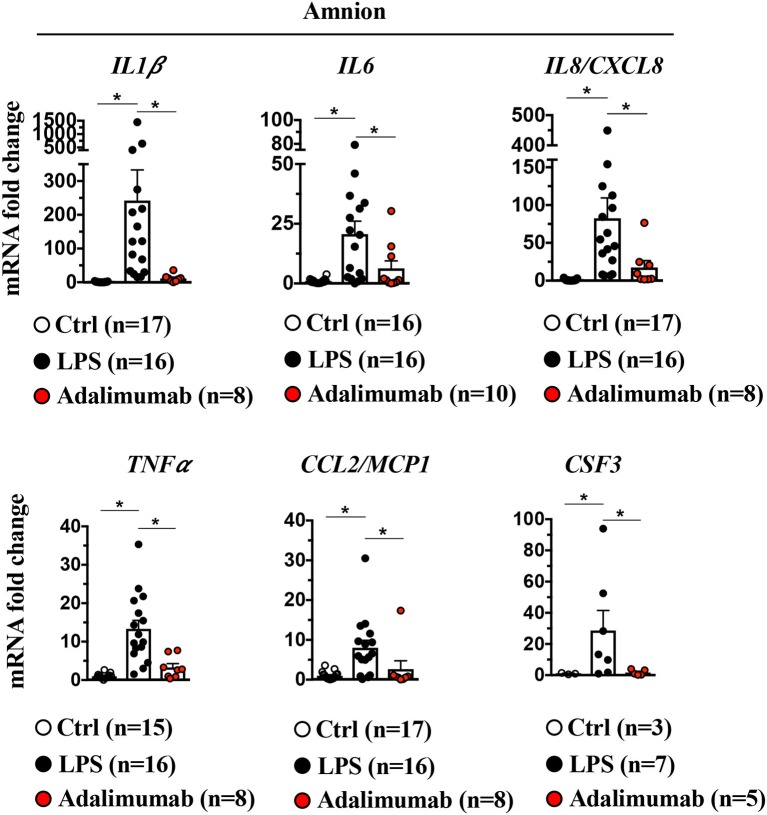
Signaling in amnion: Induction of LPS-induced pro-inflammatory cytokines and neutrophil chemoattractant is TNF-dependent. Amnion was physically separated from chorion and decidua parietalis, and expression of cytokine mRNAs by quantitative PCR (Taqman probes) is shown. Intraamniotic LPS induced IL1β, IL6, IL8/CXCL8 TNFα, CCL2/MCP1, and CSF3. All LPS-induced cytokine/chemokine mRNAs were inhibited by Adalimumab. Average mRNA values are fold increases over the average value for control after internally normalizing to the housekeeping 18S RNA (Ctrl *n* = 3–17; LPS *n* = 7–16; Adalimumab *n* = 5–10). Data are mean ± SEM, ^*^p < 0.05 between comparators (Mann–Whitney *U*-test).

It is unknown which of the resident tissues/cells at the maternal-fetal interface are responsible for neutrophil recruitment. Recently, we showed that the amnion, the tissue in contact with the amniotic fluid participates in recruiting neutrophils upon LPS injection into the amniotic fluid (10). Herein, we confirmed and expanded our previous results showing large increases in cytokine mRNAs including neutrophil chemotactic factors *IL8/CXCL8* and *CSF3* detected upon LPS-exposure in the amnion (Figure 6). Adalimumab significantly decreased LPS-induced increases in inflammatory mediators in the amnion (Figure 6).

### Adalimumab Blocks Inflammation in Amniotic Fluid, but Not in the Uterus

**Figure 7:**
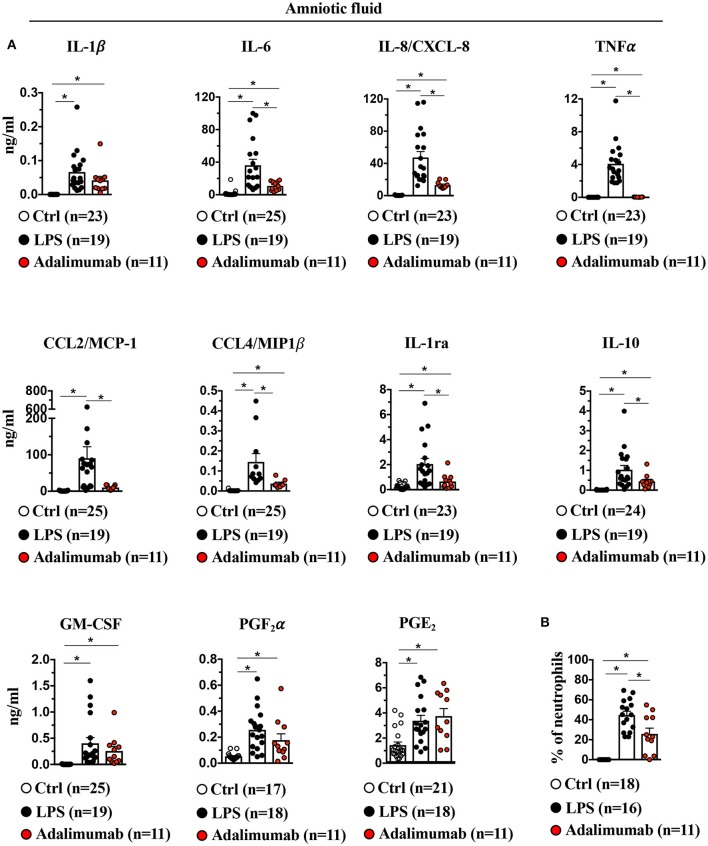
Adalimumab decreased intrauterine inflammation in amniotic fluid. **(A)** Cytokine and prostaglandins concentrations were measured in amniotic fluid by multiplex ELISA. IL-1β, IL-6, IL-8/CXCL8, TNFα, CCL2/MCP1, CCL4/MIP1β, IL-1ra, IL-10, but not GM-CSF, PGF2α and PGE2 were significantly decreased upon Adalimumab treatment. **(B)** Adalimumab decreased frequency of neutrophils in amniotic fluid. Data are mean ± SEM, ^*^p < 0.05 vs. controls (Ctrl *n* = 17–25; LPS *n* = 16–19; Adalimumab *n* = 11; Mann–Whitney *U*-test).

IA LPS-exposure significantly increased pro-and anti-inflammatory cytokines (IL-1β, IL-6, TNFα, IL-1ra, IL-10, and GM-CSF), chemokines (IL-8/CXCL-8, CCL2/MCP-1, and CCL4/MIP1β) and prostaglandins (PGF_2_α and PGE_2_) in the amniotic fluid (AF) (Figure 7A). LPS-exposure also significantly increased the frequency of neutrophils in AF (Figure 7B). Adalimumab broadly decreased LPS-induced increases in pro- and anti-inflammatory cytokines in the AF except IL-1β and GM-CSF (Figure 7A). TNF-blockade also decreased the frequency of neutrophils in AF (Figure 7B), but did not decrease LPS-induced increases in prostaglandins in the AF (Figure 7A).

LPS increased mRNAs for *IL1*β (150-fold), *IL8* (100-fold), IL6 (50-fold), *TNF*α (100-fold), and *CCL2* (100-fold) in the uterus (Supplementary Figure 11). The uterine abundance of contraction associated gap junction protein Connexin-43 (*GJA1*) mRNA also increased after LPS (40-fold) in the uterus (Supplementary Figure 11). In contrast to effects in the fetal membranes and AF, TNF blockade did not decrease mRNA for inflammatory mediators or Connexin-43 in the uterus (Supplementary Figure 11), suggesting that effect of Adalimumab is limited to the feto-maternal interface.

### Adalimumab Partially Blocked LPS-Induced Cytokine Increases in Fetal Plasma

**Figure 8:**
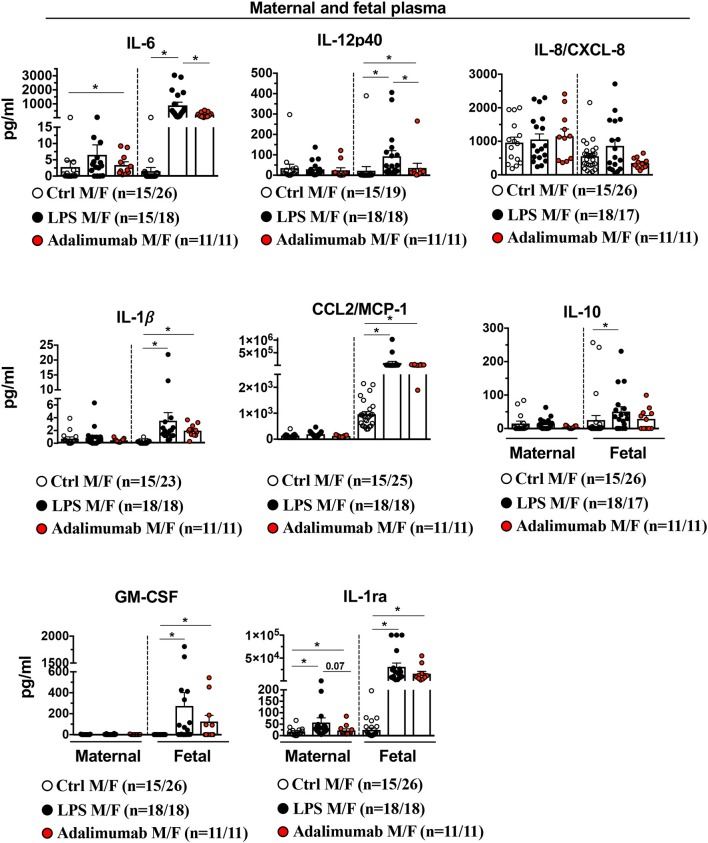
Adalimumab decreased partially inflammation in fetal plasma. Cytokine concentrations were measured in maternal and fetal plasma by multiplex ELISA. There was a minimal increase in cytokines in maternal plasma after IA LPS. In the fetal plasma, Adalimumab significantly decreased LPS-induced IL-6, and IL12p40, but not IL-8/CXCL8, IL-1β, CCL2/MCP1, IL10, GM-CSF, and IL-1ra (Maternal plasma: Ctrl *n* = 15; LPS *n* = 15–18; Adalimumab *n* = 11; Fetal plasma: Ctrl *n* = 19–26; LPS *n* = 17–18; Adalimumab *n* = 11). M, Maternal; F, Fetal. Data are mean ± SEM, ^*^p < 0.05 between comparators by Mann-Whitney *U*-test.

Consistent with a lack of significant changes in the gene expression profiles of maternal blood neutrophils in the various experimental groups, the inflammation was almost entirely localized to the intrauterine compartment since there were no changes in plasma cytokines of dams injected with IA LPS (except modest increases in plasma IL-6 and IL-1ra), and plasma cytokines during IUI were ~3-log fold lower (Figure 8) compared to AF values. In contrast to maternal blood, fetal plasma levels for IL-6, IL-12p40, IL-1β, CCL2/MCP1, IL-10, GM-CSF, IL-1ra, but not IL-8/CXCL8 increased significantly with LPS-exposure (Figure 8) and Adalimumab significantly decreased LPS-induced IL-6 and IL-12p40 levels, but did not change IL-1β, IL-8, CCL2/MCP1, and IL1-ra with non-significant decreases in GM-CSF and IL-10 levels (Figure 8).

## Discussion

The intrauterine space at the maternal-fetal interface has several compartments: fetal membranes (comprising amnion, chorion, decidua parietalis), amniotic fluid, uterus, and the placenta (comprising villous placenta, decidua basalis, and amnion-chorion). Although the fetal membranes are recognized to play an important role in IUI (24), mechanisms of immune cell activation in different intrauterine compartments during infection are poorly understood. Using our previously characterized model of IUI in preterm pregnant rhesus macaques (8, 10). We now demonstrate that inhibition of TNF-signaling reduces LPS-induced inflammation in all the intrauterine sub-compartments with the exception of the uterus. The phenotype as well as the function of neutrophils, the most abundant immune cells at the maternal-fetal interface during IUI, has not been well-characterized. Our results identify differences in gene expression profiles between neutrophils found in maternal blood and those residing in the chorio-decidua at baseline. Furthermore, in the setting of IUI chorio-decidua neutrophils acquire a classic pro-inflammatory gene expression profile (25) that is largely TNF-signaling dependent. In summary, these studies improve our understanding of the role of TNF-signaling plays in mediating IUI and neutrophil regulation.

Among the different causes of preterm labor (PTL), IUI is the best characterized both clinically and experimentally (3). Although in our model PTL was not observed in any animal, our focus was to identify early mechanisms of IUI that may subsequently lead to PTL and hence we used a short LPS-exposure interval of 16 h. In catheterized rhesus macaques, PTL was reported with a variety of stimuli both from Gram negative and positive organisms within 3d exposure (26, 27). However, in our model we did not observe PTL with IA LPS even after a 5day exposure despite a higher LPS dose than what has been reported (Kallapur, 2019, unpubl. data). The observed peak AF levels of TNFα and IL-6 in our studies were similar to previously published data, however the PGF2α and IL-1β levels were lower compared to the Boldenow et al. (27) and Adams Waldorf et al. (26) studies. Thus, the discrepancy in the observed results, presence or absence of preterm labor, may be due to the differences in the experimental animal model (e.g., indwelling catheters in the previous study compared to no instrumentation in our study) or the net lower load of some inflammatory mediators in our study. According to a multi-center biomarker study of IUI, when compared to human data, the IL-6 levels in the AF in our study would be categorized as “severe inflammation” (28). Contrary to our data, studies in mice did not show a benefit to neutrophil depletion in protecting against LPS induced PTL (29, 30). However, these mice had increased systemic concentrations of myeloid hematopoietic progenitors and myelopoietic cytokines G-CSF, IL-23, and IL-17 (31), presumably due to a “neurostat” mechanism sensing neutrophil depletion. Clinical human studies clearly definitively demonstrate that IUI increases the risk for PTL, however PTL is not an invariable consequence of IUI (28).

Leukocytes comprise about 70% of all chorio-decidua cells in the third trimester in both humans and rhesus macaques (10). During LPS-induced IUI, neutrophils are the immune subtype infiltrating the chorio-decidua and the amniotic fluid. Indeed, there were no changes in the frequency of T cell, NK cells, iNKT cells in the chorio-decidua after LPS in our model. Recently, Tong et al. reported that induction of neutrophil activation and Neutrophil Extracellular Trap (NET) release is mediated at least in part by fetal membrane-derived TNFα (11). We observed that the inhibition of TNF-signaling decreased LPS-induced neutrophil counts by 80% in the chorio-decidua and by 50% in the amniotic fluid. Based on our SNP analyses, chorio-decidua neutrophils are almost entirely of maternal origin. Prior studies addressing this question used X and Y chromosome differences between mother and male fetuses. Notably, our results using divergence in SNPs as a methodology allowed us to determine the genetic origin of neutrophils regardless of fetal sex. Despite the differences in methodology, our results are consistent with previous studies demonstrating that neutrophils infiltrating the chorio-decidua are largely of maternal origin (32, 33). This is in contrast to the neutrophils in the amniotic fluid that are largely of fetal origin (34), or mixed fetal/maternal origin (35).

In addition to inhibiting neutrophil recruitment, TNF-blockade decreased LPS-induced production of reactive oxygen species, CD63/CD16 expression on neutrophils. CD63 is critical to processing and secretion of neutrophil elastase (21). CD16 expression is induced by TNFα and mediates phagocytosis of immune complexes (22). We previously reported that chorio-decidua neutrophils are a major source of TNFα and CXCL8/IL-8 at the maternal-fetal interface (8, 10) and placentally-derived IL-8 efficiently activates neutrophils and triggers NET formation in a paracrine manner (36). Thus, our results demonstrate that TNF-signaling mediates both neutrophil recruitment and a broad program of neutrophil activation at the maternal-fetal interface.

Compared to the circulating neutrophils, those found in the chorio-decidua under homeostatic conditions had a higher expression of genes mediating extracellular matrix organization, angiogenesis, cell migration, and regulation of apoptotic processes and lower expression of genes involved in the inflammatory pathways such as TLR signaling and type I interferon signaling pathways. These findings are in line with recent data showing striking differences between the transcriptome of choriodecidua and maternal circulating leukocytes (37). Although the results seem counter-intuitive to the generally held notion that tissue neutrophils are pro-inflammatory, placenta neutrophils can sub-serve homeostatic functions and several studies report regulatory functions for these neutrophils (38–40). Consistent with increased *VEGFA* expression in the neutrophils from chorio-decidua compared to blood in our study, other authors reported pro-angiogenic neutrophils in normal 1st and 2nd trimester human decidua basalis (41, 42). Based on peak neutrophil influx after the onset of normal labor, post-partum uterus/decidua matrix remodeling and wound healing function has been attributed to decidua neutrophils (13, 43–45). Thus, our data support the concept of neutrophil heterogeneity and a role in tissue homeostasis at the maternal-fetal interface similar to that for other tissues (25, 46).

Two major changes were evident in chorio-decidua neutrophils during LPS-induced IUI. First, neutrophil numbers increased ~10-fold becoming the most abundant decidua cell-type. The gene expression profile within neutrophils also changed from homeostatic to a pro-inflammatory phenotype, consistent with a massive accumulation of activated neutrophils at the maternal-fetal interface. Secondly, inhibition of TNF-signaling in the chorio-decidua neutrophils was associated with global changes in gene-expression during IUI. Of the 325 LPS-induced genes (>4-fold), approximately 80% were down-regulated by Adalimumab. Major LPS-induced pathways (*representative gene*) that were downregulated by Adalimumab included NFkB signaling (*IL6*), TLR-signaling (*IL1*), oxidative-burst (*MPO*), and neutrophil mediated innate immunity (*LCN2*). LPS-induced genes not affected by Adalimumab include negative regulators of cytokine signaling (*SOCS3, CISH*). Of note, these results were validated by qPCR of selected key genes. Recently, Tamassia et al. reported that the use of Adalimumab decreased the production of IL-23 protein as well as decreased levels of IL12B and IL23A transcripts in TLR-8 activated neutrophils (47). Indeed, the gene-expression profiling predicts a net effect of potent anti-inflammatory action by Adalimumab.

Consistent with our neutrophil-specific gene expression data, Adalimumab decreased LPS-induced increases in the AF levels of IL-6 and other cytokines; chorio-decidua mRNAs expression of cytokines/chemokines; and fetal plasma IL-6 and IL-12p40 levels. However, AF levels of prostaglandins (PGs) and uterus tissue mRNAs cytokines were unchanged. IL-6 and PGs are validated biomarkers of inflammation that mediate preterm labor (48–50). In rodent models of IUI that involve neutrophil influx into the placenta, TNF-blockade decreased adverse pregnancy outcomes (51, 52). Consistent with this, a small human study demonstrated that TNF-blockade improves pregnancy outcomes in women with recurrent spontaneous abortions (53). Thus, TNF-signaling appears to be important in mediating IUI triggered by multiple different etiologies. Furthermore, in our current study, decreased inflammatory signaling in the neutrophils was associated with a reduction of inflammation in multiple maternal-fetal compartments with the exception for the uterus where TNF-blockade did not decrease LPS-induced inflammation. The reasons for lack of efficacy in uterus is not clear but possibilities include uterus inflammation being regulated by other mediators such as IL-6. It is also possible that the kinetics of inflammation in the uterus may be different and our study evaluating only one time point of exposure may have missed any beneficial effects.

Other limitations of our study also have to be considered. Firstly, although in our model of IUI neutrophils represent the majority of leukocytes at the feto-maternal interface, we did not investigate the contribution of other cells to the inflammatory process, in particular macrophages. In fact, we have previously shown that TNFα producing decidua macrophage numbers do increase during IUI (10). In another recent study, Doster et al. demonstrated that in response to GBS infection placental macrophages can produce extracellular traps, similar to the NETs produced by neutrophils (54). Furthermore, other cytokines and cells (both immune cells and resident cells) may play an important role in the setting of IUI. Secondly, we did not observe preterm labor/birth in our study. TNF-inhibition may not dampen inflammation induced by live organisms including GBS or conditions that may cause PTB. Thirdly, the route of injection of Adalimumab (i.e., s.c. + IA) used in our model is not easily feasible in the clinical setting and it will be important to understand whether subcutaneous administration alone can dampen the inflammation.

**Figure 9:**
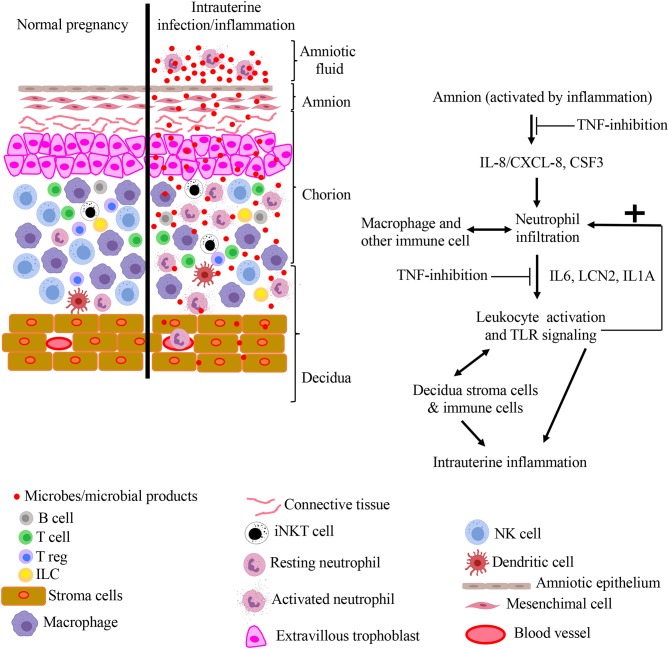
Model for pathogenesis of intrauterine infection/inflammation. Representative cells in the different tissue layers of fetal membrane are shown. The left panel in figure depicts normal pregnancy and the right panel shows changes during IUI. Inflammatory products and microbial products (red dots) in the amniotic fluid and chorio-decidua activate the amnion, resulting in the release of leukocyte chemoattractants in a TNF-dependent manner. Based on a previous study, IL-1 signaling also inhibits multiple pathways in IUI (10). Leukocytes accumulate at chorio-decidua junction, get activated, and greatly amplify the inflammation at the maternal-fetal interface possibly interacting with each other and resident cells.

Amnion epithelium is strategically located at the maternal-fetal interface and due to its contact with the AF, it is in an ideal position to sense inflammatory mediators. We previously reported that the amnion tissue expresses neutrophil-attracting chemokines during IUI in an IL1R-IRAK1 dependent manner (10), and others have demonstrated that fetal membranes can recruit and activate neutrophils (9). Here, we show that amnion expression of neutrophil chemokines is also TNF-dependent. IL1 inhibition has overlapping results compared to TNF inhibition in the same rhesus macaque model of LPS-induced IUI (10, 11). However, a notable difference is inhibition of LPS-induced AF PGE_2_ levels with IL1-blockade but not TNF inhibition. Taken together, both IL1- signaling and TNF-signaling appear to be independently important in the IUI network induced by LPS. Additionally, in the present study, we also were able to demonstrate that the TNF inhibition specifically decreased several inflammatory pathways in the chorio-decidua neutrophils. Together with previous literature, our results suggest a model for the pathogenesis of intrauterine inflammation (Figure 9), proposing a key role for both TNF- and IL1- signaling in mediating the initiation, amplification of inflammation, and regulating recruitment and activity of neutrophils and other leukocytes at the maternal-fetal interface. The results also raise the prospect of utility of specific cytokine blockade as a potential strategy for intrauterine inflammation.

## Data Availability Statement

All original RNAseq data were deposited in the NCBI's Gene Expression Omnibus database (GEO accession: GSE145918). Other raw data supporting the conclusions of this article will be made available by the authors, without undue reservation, to any qualified researcher.

## Ethics Statement

All animal procedures were approved by the IACUC at UC Davis, California Primate Center.

## Author Contributions

PP, MC, PS, FM, MM, CJ, SM, LM, MP, AJ, CC, and SK participated in data generation. PP, MC, PS, FM, MP, AJ, CC, and SK participated in analysis and interpretation of data. AJ, CC, and SK participated in the conception and design of the study and obtained the funding. PP and SK wrote the manuscript. All authors have reviewed the manuscript and approved the final version.

### Conflict of Interest

The authors declare that the research was conducted in the absence of any commercial or financial relationships that could be construed as a potential conflict of interest.
