# Supplementary material for: TNF-Signaling Modulates Neutrophil-Mediated Immunity at the Feto-Maternal Interface During LPS-Induced Intrauterine Inflammation
Source: Front Immunol. 2020 Apr 3;11:558. doi: 10.3389/fimmu.2020.00558 (PMC7145904; doi:10.3389/fimmu.2020.00558)
Supplement: Supplementary file 1 [file Data_Sheet_1.pdf]

Supplementary Table I. Clinical characteristics of animals included in the study.

	IA or IM Saline Ctrl (IA n=24; IM n=2)	IA LPS (n=19)	IA Adalimumab (n=11)
**Maternal age, year ± SD**	10.2 ± 2.8	7.8 ± 2.9	9.9 ± 2.5
**Maternal weight (Kg)**	9.1 ± 1.5	8.7 ± 1.9	9.8 ± 1.6
**Median GA at delivery, days [range]**	131 [128-136]	131 [129-137]	131 [124-136]
**Mean birth weight, gram ± SD**	330.78 ± 30.36	322.3 ± 45.3	337 ± 65.6
**Fetal gender number (F/M)**	8/18	12/7	5/6

A total of 29 animals from Saline and LPS exposure were previously used for another study (Presicce et al., 2018).

Supplementary Table II. List of monoclonal Antibodies used to sort and phenotype chorio-decidua cells by FACS-sorter and flow cytometry.

**mAb**	**Sample**	**Manufacturer**	**Clone**	**Conjugation**
CD45	Rhesus	BDBioscience	D058-1283	PE-CF594
HLA-DR	Rhesus	Biolegend	L243	Brilliant Violet 570<br>PercPCy5.5
CD3	Rhesus	BDBioscience	SP34-2	APC-Cy7
CD14	Rhesus	Thermofisher	TUK4	Pacific Blue
CD56	Rhesus	BDBioscience	NCAM16.2	PE-Cy7
CD88	Rhesus	AbD Serotec	P12/1	Alexa Fluor 647
CD19	Rhesus	Biolegend	HIB19	Alexa Fluor 700
CD20	Rhesus	Biolegend	2H7	Alexa Fluor 700
CD16	Rhesus	BDBioscience	3G8	Alexa Fluor 700
CD63	Rhesus	Biolegend	H5C6	Pacific Blue
Live/Dead	Rhesus	Thermofisher		Aqua

Supplementary Table III. List of Rhesus probes for qPCR (ThermoFisher Scientific).

IL1β	Rh02621711_m1
IL6	Rh02789322_m1
IL8	Rh02789781_m1
MCP1/CCL2	Rh02621753_m1
TNFα	Rh02789783_m1
CSF3	Rh02825033_m1
Connexin-43	AIY9Y3J (custom)
NFKB1	Rh00765726_m1
Licpolalin2 (LCN2)	Mf04378478_g1

Supplementary Table IV. Fifteen genes further upregulated by Adalimumab.

Gene
CD248
DCN
ENSMMUG00000015202
ENSMMUG00000041841
ENSMMUG00000043350
IGF1
IGKC
MEIS1
MOB2
POSTN
SFRP4
TMTC1
ENSMMUG00000043574
STRA6
ENSMMUG00000046591

Refer to Figure 2E of the main manuscript for definition of LPS-induced TNF-upregulated genes.

Supplementary Table V. TNF-dependent biological process and associated genes.

Term	P-value	Genes
inflammatory response (GO:0006954)	6.82544E-10	TNFAIP6;CCL20;PTGER2;FPR2;IL1A;IL6;IL18RAP;VNN1;ADORA2A;IL2RA;CCL2;FAS;NLRP3;PROK2;TNFRSF8;ACKR1;S100A9;S100A8;PTGDR
response to lipopolysaccharide (GO:0032496)	7.90166E-07	CD274;ADAM17;IL6;TNIP1;TNIP3;CCL2;FAS;NLRP3;TNFRSF8;IRAK3;CD14;PDCD1LG2
neutrophil mediated immunity (GO:0002446)	1.85716E-08	FCER1G;TNFAIP6;CD300A;MCEMP1;FPR2;GPR84;MMP8;C3;PLAC8;TRPM2;ADAM17;IL6;CLEC4D;PNP;VNN1;PLAU;LCN2;CTSH;S100A12;STOM;CD14;S100A9;S100A8;CD177
neutrophil degranulation (GO:0043312)	2.52604E-07	FCER1G;TNFAIP6;CD300A;MCEMP1;FPR2;GPR84;MMP8;C3;PLAC8;TRPM2;CLEC4D;PNP;VNN1;PLAU;LCN2;CTSH;S100A12;STOM;CD14;S100A9;S100A8;CD177
neutrophil activation involved in immune response (GO:0002283)	2.90962E-07	FCER1G;TNFAIP6;CD300A;MCEMP1;FPR2;GPR84;MMP8;C3;PLAC8;TRPM2;CLEC4D;PNP;VNN1;PLAU;LCN2;CTSH;S100A12;STOM;CD14;S100A9;S100A8;CD177
neutrophil chemotaxis (GO:0030593)	6.26626E-05	FCER1G;CCL20;CCL2;S100A12;S100A9;S100A8
neutrophil migration (GO:1990266)	9.77921E-06	FCER1G;CCL20;CCL2;S100A12;S100A9;S100A8;CD177
cytokine-mediated signaling pathway (GO:0019221)	1.18183E-08	CSF3;IL1RN;EBI3;IL2RG;CSF2RA;TANK;ICAM1;IL18RAP;IRAK2;CCL2;HMOX1;TNFRSF8;FCGR1A;IL13RA2;IL4R;CCL20;IRAK3;RTN4RL2;SOD2;BATF;IL1A;ADAM17;IL6;IL23A;IL2RA;LCN2;FAS;IL18R1
positive regulation of cytokine production (GO:0001819)	2.83507E-05	C3;IL1A;ADAM17;IL6;IL23A;CLEC6A;HILPDA;FLOT1;NLRP3;CD14;TANK;ADRA2A
cellular response to cytokine stimulus (GO:0071345)	4.58696E-07	CSF3;IL1RN;IL4R;CCL20;IRAK3;RTN4RL2;IL2RG;CSF2RA;TANK;BATF;ICAM1;IL1A;IL6;IL23A;IRAK2;IL2RA;LCN2;CCL2;HMOX1;IL13RA2;IL18R1
interleukin-18-mediated signaling pathway (GO:0035655)	0.004310026	IL18RAP;IL18R1
apoptotic process (GO:0006915)	4.55578E-05	IL1A;PLSCR1;ADORA2A;CASP5;IL2RA;FAS;NLRP3;PLAGL2;CD14;CFLAR;BCL2L14;IER3
regulation of extrinsic apoptotic signaling pathway via death domain receptors (GO:1902041)	0.000148041	FAS;HMOX1;CFLAR;TANK;ICAM1
toll-like receptor signaling pathway (GO:0002224)	1.66812E-06	TNIP1;IRAK2;TNIP3;IRAK3;TLR10;CD14;PIK3AP1;S100A9;S100A8
toll-like receptor 4 signaling pathway (GO:0034142)	0.001014465	TNIP3;CD14;PIK3AP1
pattern recognition receptor signaling pathway (GO:0002221)	3.57815E-05	IRAK2;IRAK3;TLR10;CD14;S100A9;S100A8

Similar biological processes are grouped by the same color. Refer to Figure 2E of the main manuscript for definition of TNF-dependent gene expression.

Supplementary Table VI. Coverage of the representative SNPs.

Ctrl #427	Nucleotide	chr14: 61,428,027 (TRIM22)	chr14: 61,440,616 (TRIM22)	chr13: 29,720,032 (FOSL2)	chr13: 28,721,094 (FOSL2)	chr13: 28,721,275 (FOSL2)
Fetal Lung	A	8 (44%)	0	0	0	10 (100%)
	C	0	10 (45%)	29 (100%)	12 (100%)	0
	G	10 (56%)	0	0	0	0
	T	0	12 (55%)	0	0	0
Chorio-Decidua Neutrophils	A	0	0	0	0	88 (48%)
	C	0	0	132 (54%)	315 (51%)	0
	G	57 (100%)	0	0	306 (49%)	97 (52%)
	T	0	43 (100%)	111 (46%)	0	0
Maternal Blood Neutrophils	A	0	0	0	0	12 (52%)
	C	0	0	215 (46%)	30 (48%)	0
	G	519 (100%)	0	0	33 (52%)	11 (48%)
	T	0	147 (100%)	255 (54%)	0	0

LPS #442	Nucleotide	chr19: 44,875,344 (RPL13A)	chr14: 61,428,027 (TRIM22)	chr14: 61,428,690 (TRIM22)	chr6: 130,173,358 (IRF1)	chr13: 28,701,305 (FOSL2)
Fetal Lung	A	145 (63%)	36 (100%)	36 (100%)	166 (75%)	34 (48%)
	C	0	0	0	0	37 (52%)
	G	0	0	0	0	0
	T	85 (37%)	0	0	56 (25%)	0
Chorio-Decidua Neutrophils	A	67 (100%)	33 (49%)	17 (59%)	80 (100%)	0
	C	0	0	0	0	151 (100%)
	G	0	35 (51%)	12 (41%)	0	0
	T	0	0	0	0	0
Maternal Blood Neutrophils	A	124 (99%)	191 (53%)	116 (58%)	379 (100%)	0
	C	0	0	0	0	54 (100%)
	G	0	169 (47%)	83 (42%)	0	0
	T	1 (1%)	0	0	0	0

Adalimumab + LPS #431	Nucleotide	chr6: 130,172,806 (IRF1)	chr2: 148,997,781 (SF3B1)	chr1: 130,601,437 (SSR2)	chr14: 61,430,065 (TRIM22)	chr1: 126,603,427 (S100A11)
Fetal Lung	A	28 (100%)	0	41 (58%)	0	0
	C	0	35 (100%)	0	11 (100%)	0
	G	0	0	30 (42%)	0	72 (100%)
	T	0	0	0	0	0
Chorio-Decidua Neutrophils	A	46 (42%)	0	39 (100%)	0	120 (48%)
	C	0	17 (55%)	0	15 (48%)	0
	G	64 (58%)	0	0	0	130 (52%)
	T	0	14 (45%)	0	16 (52%)	0
Maternal Blood Neutrophils	A	32 (42%)	0	13 (100%)	0	79 (51%)
	C	0	15 (56%)	0	128 (42%)	0
	G	44 (58%)	0	0	0	75 (49%)
	T	0	12 (44%)	0	180 (58%)	0

The number represents the number of reads that overlap the SNP with the specific nucleotide. Numbers in brackets represent the frequency of nucleotides at each of the informative SNPs (refer to Figure 4F of the main manuscript).
