# Supplementary material for: TNF-Signaling Modulates Neutrophil-Mediated Immunity at the Feto-Maternal Interface During LPS-Induced Intrauterine Inflammation
Source: Front Immunol. 2020 Apr 3;11:558. doi: 10.3389/fimmu.2020.00558 (PMC7145904; doi:10.3389/fimmu.2020.00558)
Supplement: Supplementary file 2 [file Image_1.pdf]

Supplementary Figure 1.

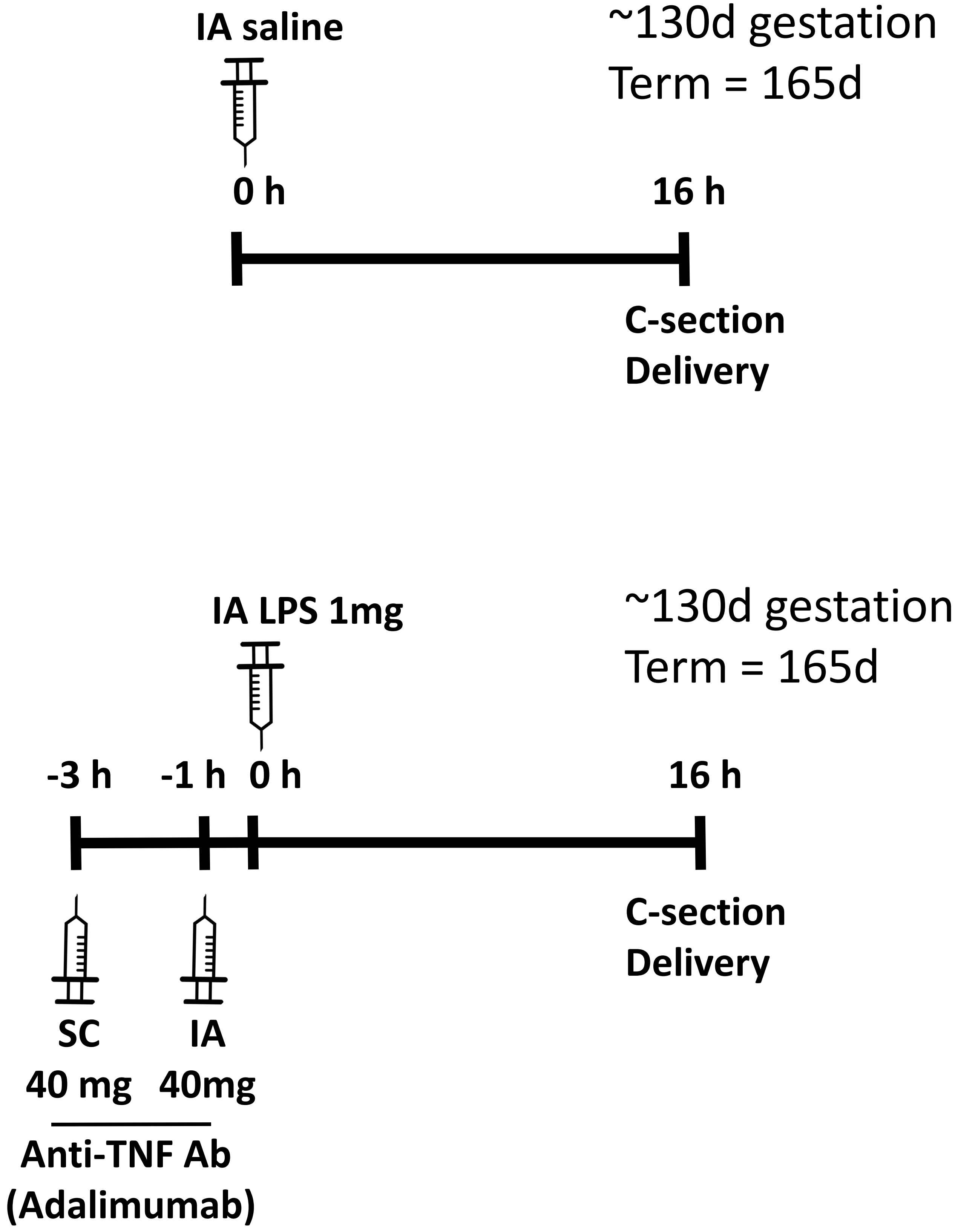

Supplementary Figure 1. Experimental design of saline and Adalimumab treatment in LPS-exposed animals.
