# Supplementary material for: TNF-Signaling Modulates Neutrophil-Mediated Immunity at the Feto-Maternal Interface During LPS-Induced Intrauterine Inflammation
Source: Front Immunol. 2020 Apr 3;11:558. doi: 10.3389/fimmu.2020.00558 (PMC7145904; doi:10.3389/fimmu.2020.00558)
Supplement: Supplementary file 3 [file Image_2.pdf]

Supplementary Figure 2.

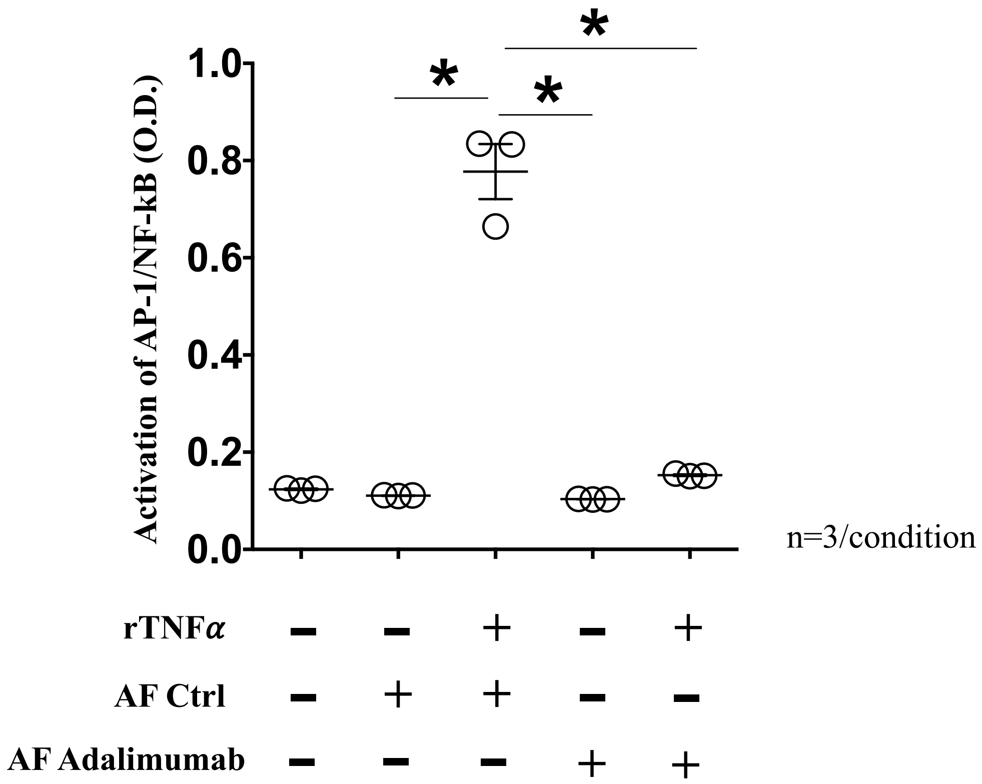

Supplementary Figure 2. Adalimumab specifically inhibits TNFα. HEK-Dual TNFα cells were stimulated *in vitro* o.n. with or without rTNFα (4 ng/ml) and/or amniotic fluid (AF, dilution 1:10) obtained at delivery from Ctrl or Adalimumab animals with the read-out of activation of AP-1/NF-kB. As expected, HEK-Dual TNFα cells did not respond to exogenous LPS (not shown). When HEK-Dual TNFα cells were cultured with rTNFα alone or rTNFα + AF from Ctrl animals, the activation of AP-1/NF-KB was significantly increased compared to baseline. In contrast, the presence of AF from Adalimumab injected animals reversed the activation of rTNFα stimulated AP-1/NF-kB to baseline levels, demonstrating specific TNFα inhibitory activity at the time-point when assays for neutrophil activity were performed. . Detection of bioactive TNFα is evaluated by the activation of the AP-1/NF-kB pathway. n=3/condition. P * <0.05 (Unpaired t test).
