# Supplementary material for: TNF-Signaling Modulates Neutrophil-Mediated Immunity at the Feto-Maternal Interface During LPS-Induced Intrauterine Inflammation
Source: Front Immunol. 2020 Apr 3;11:558. doi: 10.3389/fimmu.2020.00558 (PMC7145904; doi:10.3389/fimmu.2020.00558)
Supplement: Supplementary file 4 [file Image_3.pdf]

Supplementary Figure 3.

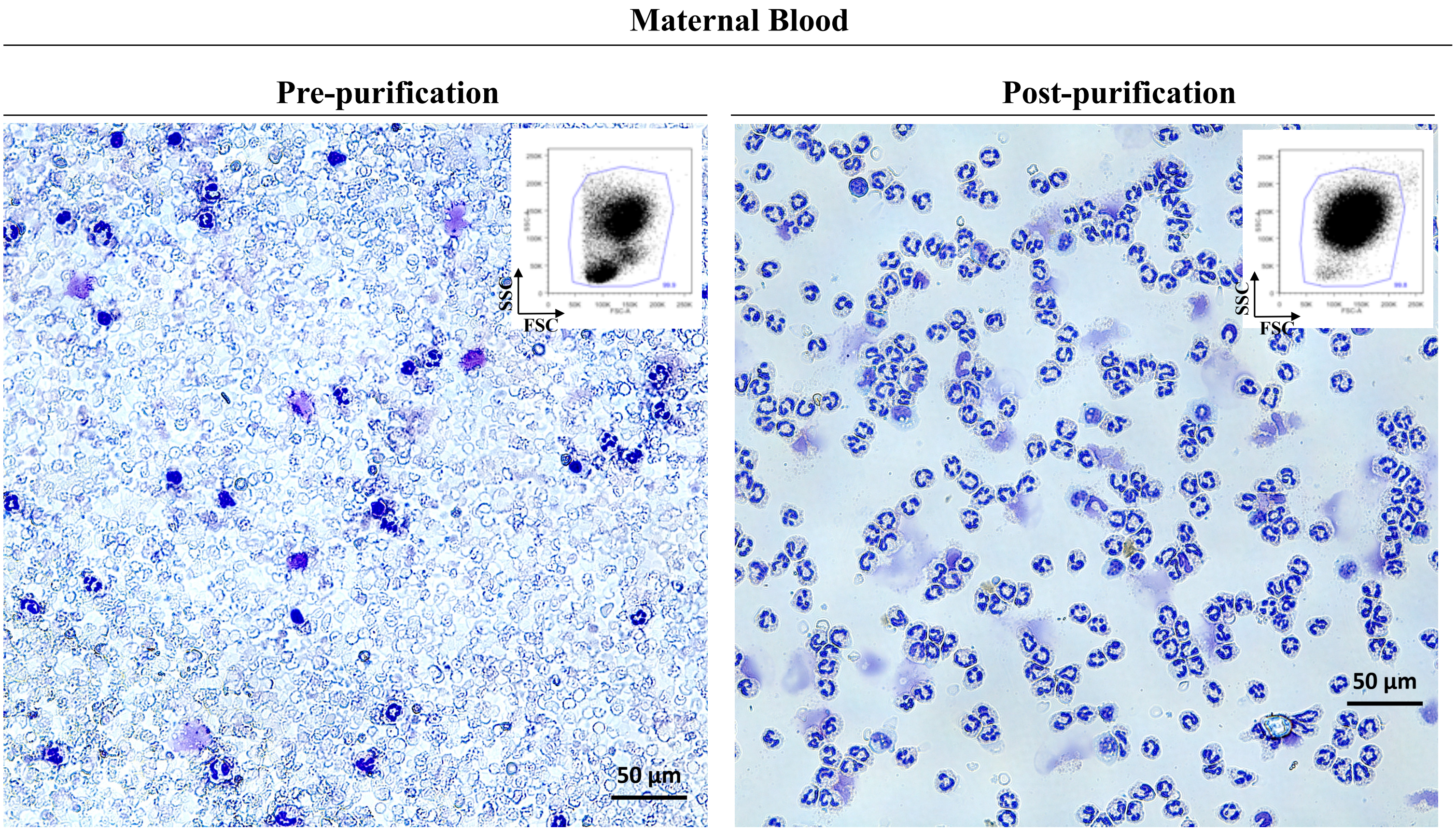

Supplementary Figure 3. Maternal blood neutrophils were obtained by magnetic bead isolation with a purity >97%. Representative dot plots (n=3, upper right) and Diff-quick staining (n=3) showing pre- and post-purification of maternal peripheral blood neutrophils. The integrity of purified total RNA from bead-isolated maternal blood neutrophils (10-50 ng per sample) was assessed using HighSensitivity RNA ScreenTapes on the TapeStation 2200 (Agilent Technologies).
