# Supplementary material for: TNF-Signaling Modulates Neutrophil-Mediated Immunity at the Feto-Maternal Interface During LPS-Induced Intrauterine Inflammation
Source: Front Immunol. 2020 Apr 3;11:558. doi: 10.3389/fimmu.2020.00558 (PMC7145904; doi:10.3389/fimmu.2020.00558)
Supplement: Supplementary file 5 [file Image_4.pdf]

Supplementary Figure 4.

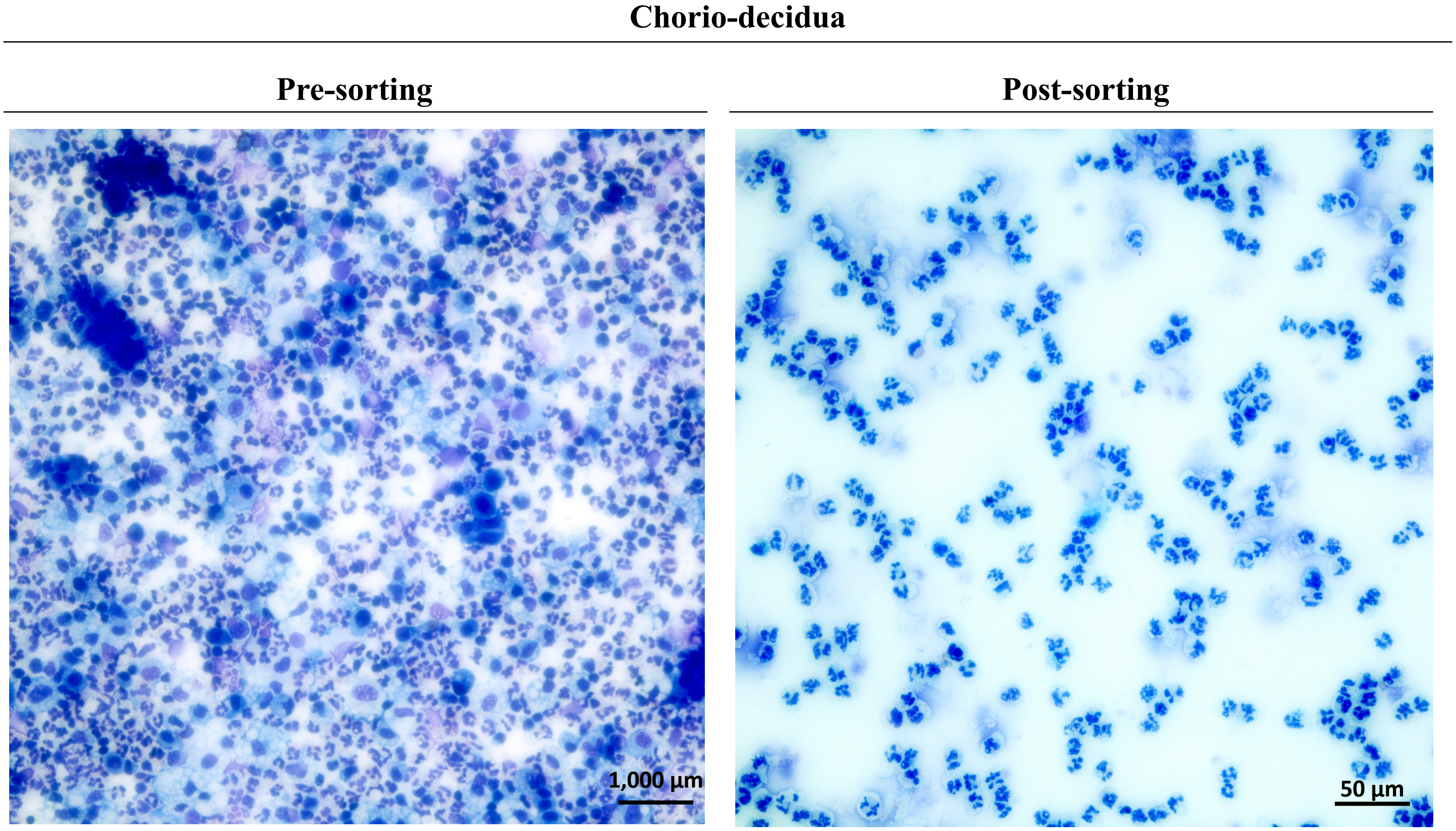

Supplementary Figure 4. Chorio-decidua neutrophils (defined as CD3$^-$CD14^{low}HLADR$^-$CD88$^+$CD56$^-$ cells) were FACS-sorted from the three groups of animals with a purity >97% (not shown). Representative (n=5) Diff-Quick staining of pre- and post-sorted cells. The integrity of purified total FACS-sorted chorio-decidua neutrophils (10-50 ng per sample) was assessed using HighSensitivity RNA ScreenTapes on the TapeStation 2200 (Agilent Technologies).
