# Supplementary material for: TNF-Signaling Modulates Neutrophil-Mediated Immunity at the Feto-Maternal Interface During LPS-Induced Intrauterine Inflammation
Source: Front Immunol. 2020 Apr 3;11:558. doi: 10.3389/fimmu.2020.00558 (PMC7145904; doi:10.3389/fimmu.2020.00558)
Supplement: Supplementary file 6 [file Image_5.pdf]

Supplementary Figure 5.

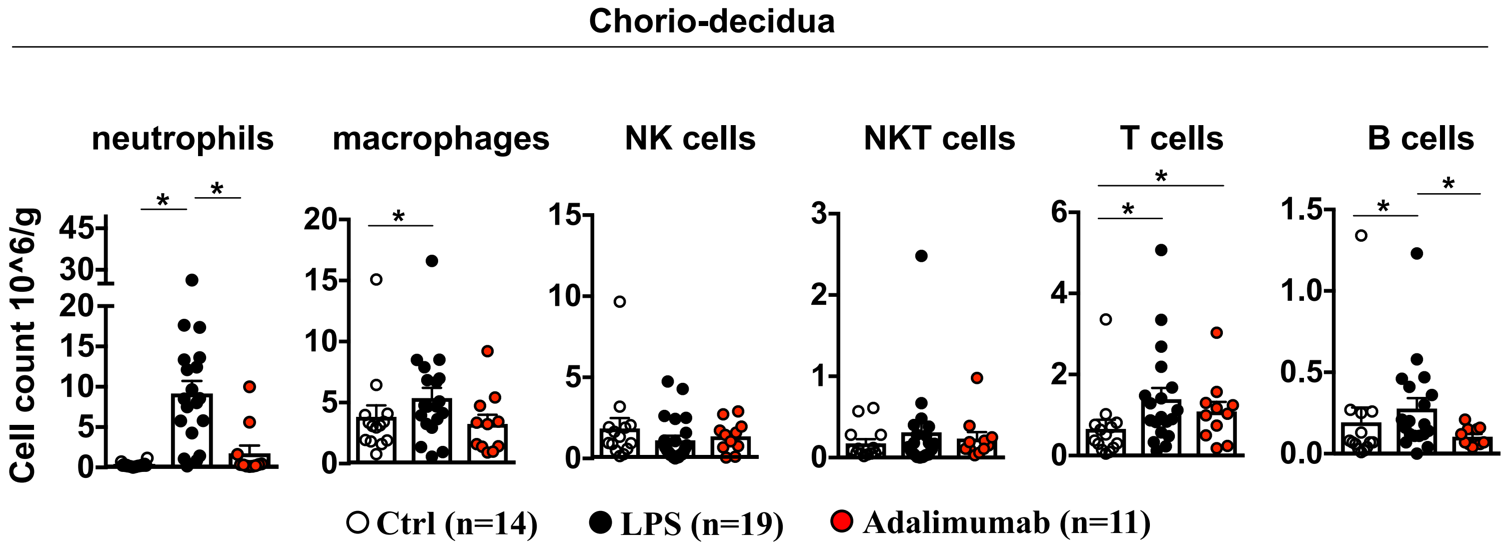

Supplementary Figure 5. Adalimumab decreased significantly neutrophil and B cell count. Chorio-decidua cell suspensions were analyzed by multiparameter flow cytometry and the different leukocyte populations were defined as previously described (Presicce et al., 2018). Briefly, live cells were first identified by the absence of LIVE/DEAD stain and forward-/side-scatter expression, excluding cell debris. Leukocytes were gated as CD45^{+} cells. IA LPS exposure increased significantly the number of chorio-decidua (CD3^{-}CD14^{low}HLADR^{-}CD88^{+}CD56^{-}) neutrophils, (CD3^{-}CD14^{high}CD88^{+}HLA-DR^{+}) macrophages, (CD14^{-}CD56^{-}CD3^{+})T cells and (CD3^{-}CD14^{-}CD56^{-}CD19/CD20^{+}) B cells compared to the control animals, but Adalimumab reverted only neutrophil and B cell numbers to control levels. LPS had no impact on (CD3^{-}CD14^{-}HLA-DR^{-}CD88^{-}CD56^{+}) NK or (CD14^{-}CD3^{+}CD56^{+} NKT) cell numbers. Cell count was expressed per gram of tissue (Ctrl n=14; LPS n=19; Adalimumab n=11). Data are mean, SEM, *p<0.05 between comparators.
