# Supplementary material for: TNF-Signaling Modulates Neutrophil-Mediated Immunity at the Feto-Maternal Interface During LPS-Induced Intrauterine Inflammation
Source: Front Immunol. 2020 Apr 3;11:558. doi: 10.3389/fimmu.2020.00558 (PMC7145904; doi:10.3389/fimmu.2020.00558)
Supplement: Supplementary file 7 [file Image_6.pdf]

Supplementary Figure 6.

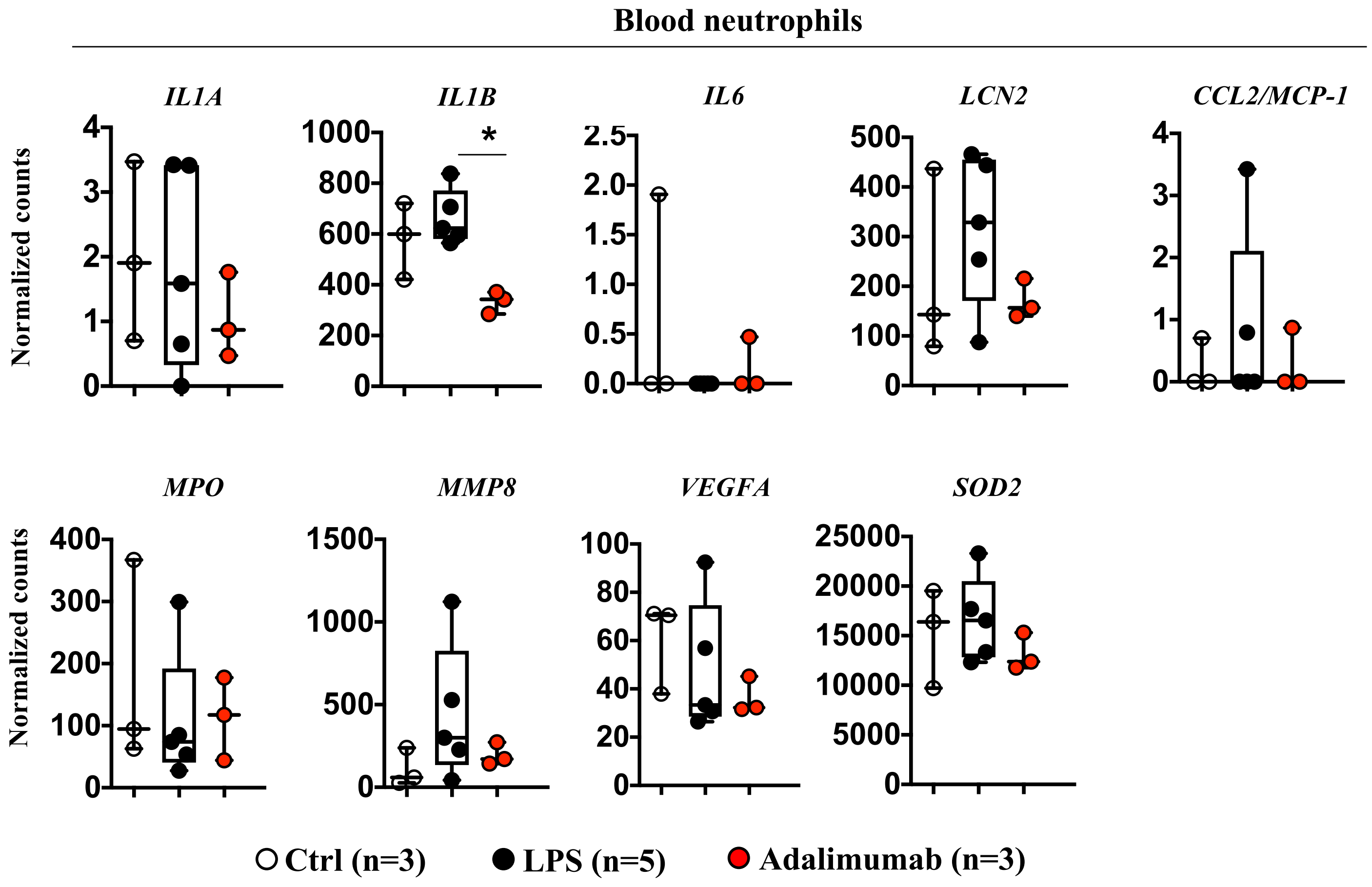

Supplementary Figure 6. Maternal blood neutrophil gene expression profile do not differ significantly after LPS or Adalimumab. RNA-seq analysis of neutrophils isolated from maternal blood using magnetic beads. Normalized counts of gene expression value of representative genes (Ctrl n=3; LPS n=5; Adalimumab n=3 for all the genes). Data are mean, SEM, *p<0.05 between comparators (Mann–Whitney U test).
