# Supplementary material for: TNF-Signaling Modulates Neutrophil-Mediated Immunity at the Feto-Maternal Interface During LPS-Induced Intrauterine Inflammation
Source: Front Immunol. 2020 Apr 3;11:558. doi: 10.3389/fimmu.2020.00558 (PMC7145904; doi:10.3389/fimmu.2020.00558)
Supplement: Supplementary file 8 [file Image_7.pdf]

Supplementary Figure 7.

A.

Blood vs. Chorio-decidua neutrophils– upregulated genes (n=3)

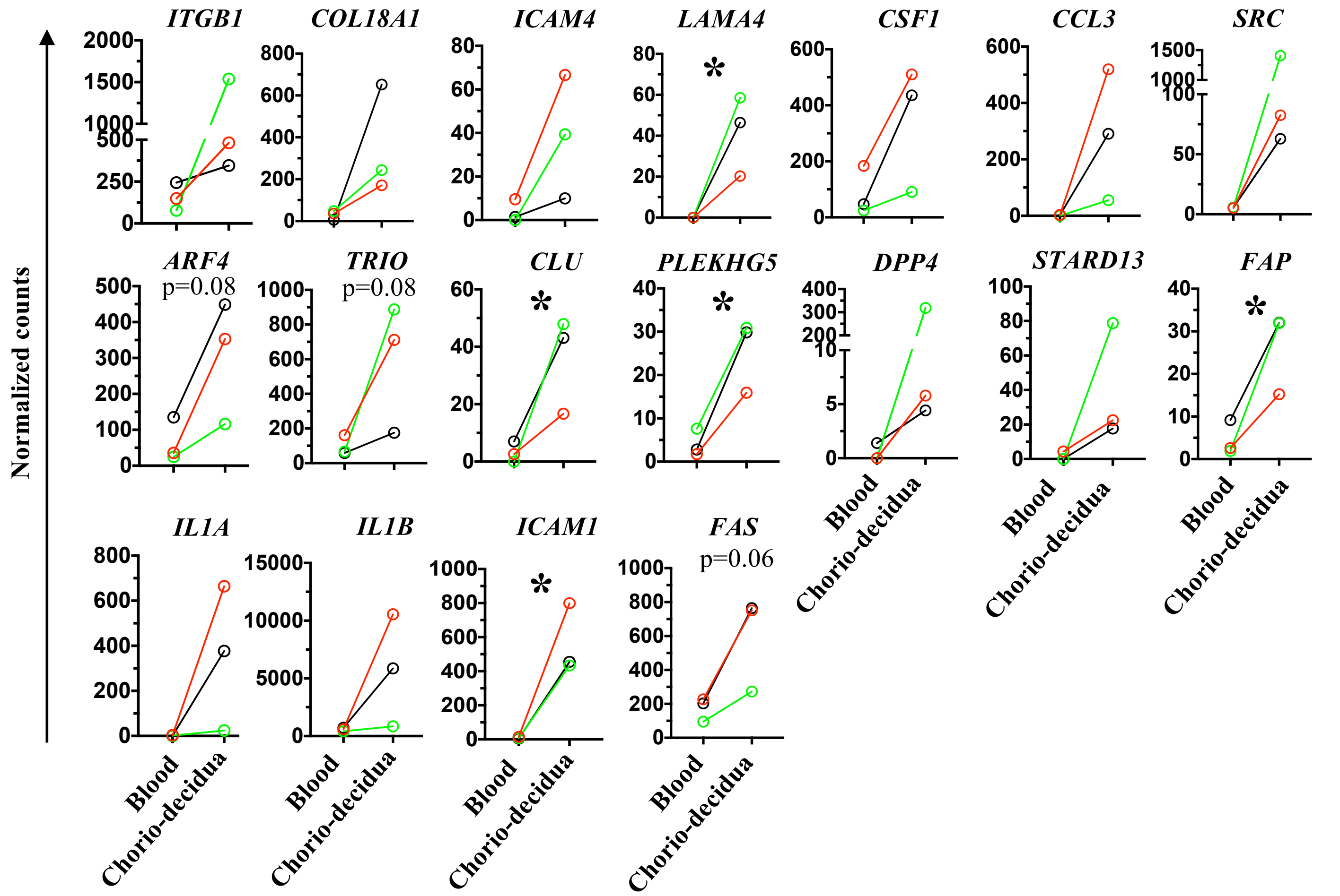

B.

Blood vs. Chorio-decidua neutrophils – downregulated genes (n=3)

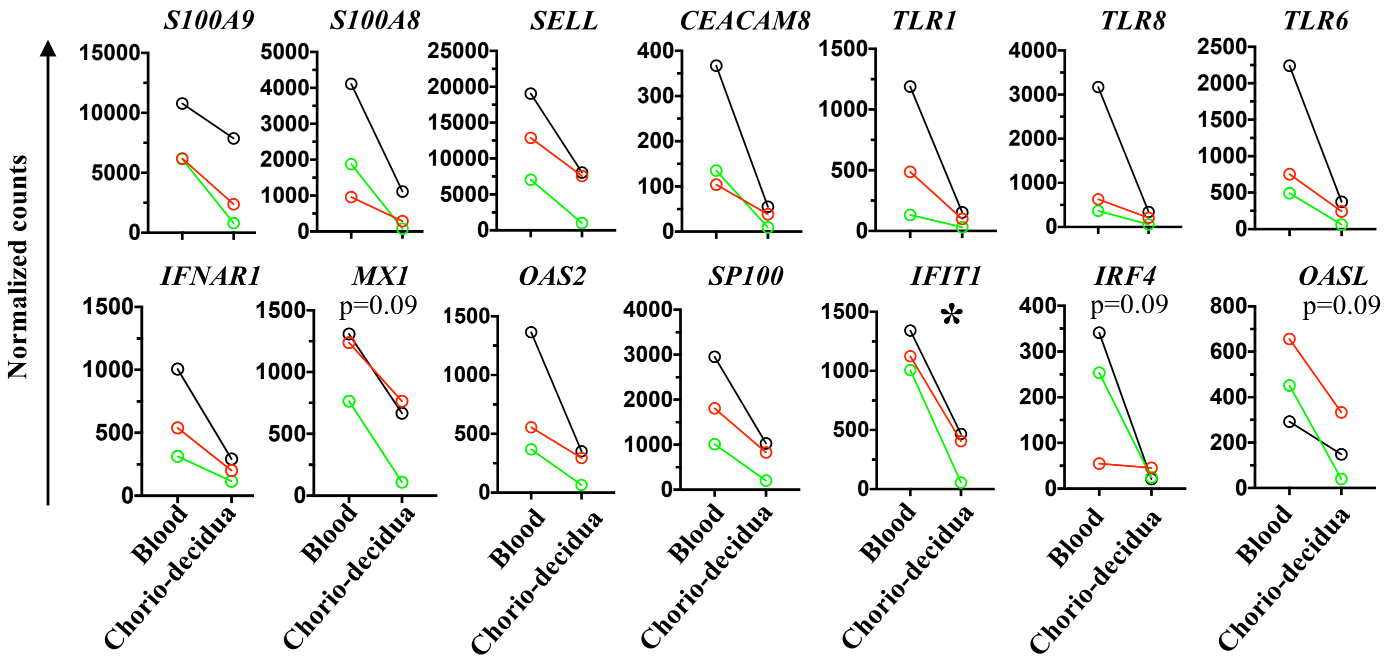

Supplementary Figure 7. Differential expression genes in blood vs. chorio-decidua neutrophils. Representative genes associated to the biological processes [see Figure 1D] **(A)** upregulated or **(B)** downregulated in chorio-decidua neutrophils compared to blood neutrophils. The lines show normalized counts of gene-expression trend plot from the same control animal (n=3: black line= sample #427, red line = sample #432, green line = sample #430). p * <0.05 (Unpaired t test).
