# Supplementary material for: TNF-Signaling Modulates Neutrophil-Mediated Immunity at the Feto-Maternal Interface During LPS-Induced Intrauterine Inflammation
Source: Front Immunol. 2020 Apr 3;11:558. doi: 10.3389/fimmu.2020.00558 (PMC7145904; doi:10.3389/fimmu.2020.00558)
Supplement: Supplementary file 9 [file Image_8.pdf]

Supplementary Figure 8 .

A.

Chorio-decidua neutrophils - TNF-dependent genes

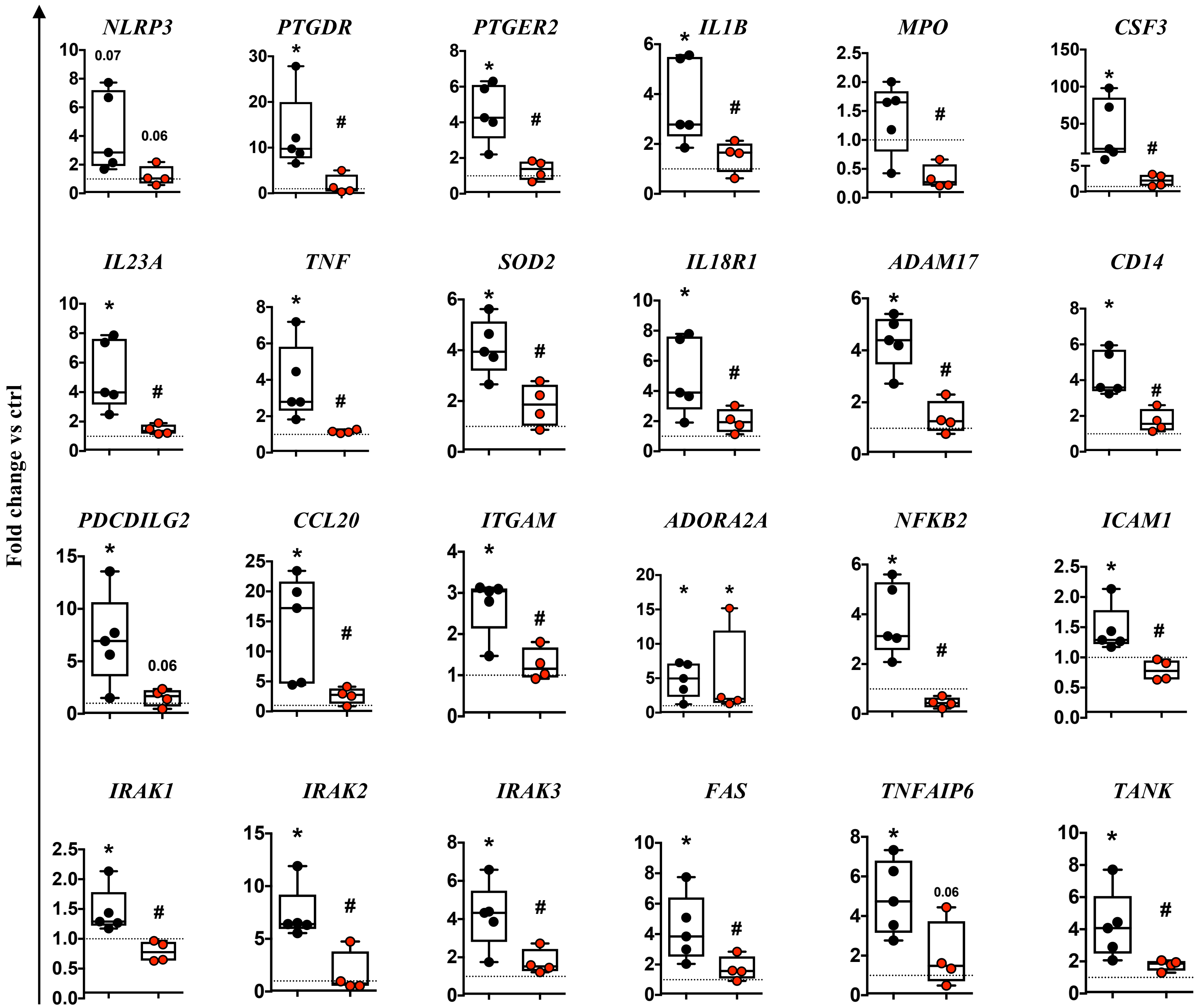

Ctrl (mean of 3) **LPS (n=5)** **Adalimumab (n=4)**

B.

Validation of selected transcriptomic data by qPCR

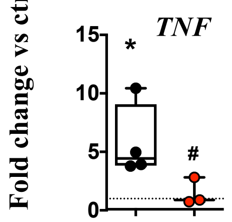

Ctrl (mean of 3) **LPS (n=4)** **Adalimumab (n=3)**

Supplementary Figure 8. Genes associated to the biological processes inhibited by Adalimumab. (A) Extended list of genes representing biological processes inhibited by Adalimumab shown in Figure 3. The box plot shows fold-change of gene expression in chorio-decidua neutrophils normalized to control chorio-decidua neutrophils (dotted-line). P * <0.05 vs. ctrl; # <0.05 vs. LPS (dotted-line represents the mean of 3 Ctrl; LPS n=5; Adalimumab n=4. Mann–Whitney U test). **(B)** Validation of selected transcript expression by qPCR analysis. mRNAs were isolated FACS-sorted chorio-decidua neutrophils from Ctrl, LPS-, and Adalimumab-treated animals. qPCR was performed using rhesus-specific Taqman probes. The values were first internally normalized to the endogenous 18S RNA, and the box plot show fold-change of gene expression normalized to control chorio-decidua neutrophils (dotted-line represents the mean of 3 Ctrl; LPS n=4; Adalimumab n=3). P * <0.05 vs. ctrl; # <0.05 vs. LPS (Mann–Whitney U test).
