# Supplementary material for: TNF-Signaling Modulates Neutrophil-Mediated Immunity at the Feto-Maternal Interface During LPS-Induced Intrauterine Inflammation
Source: Front Immunol. 2020 Apr 3;11:558. doi: 10.3389/fimmu.2020.00558 (PMC7145904; doi:10.3389/fimmu.2020.00558)
Supplement: Supplementary file 10 [file Image_9.pdf]

Supplementary Figure 9.

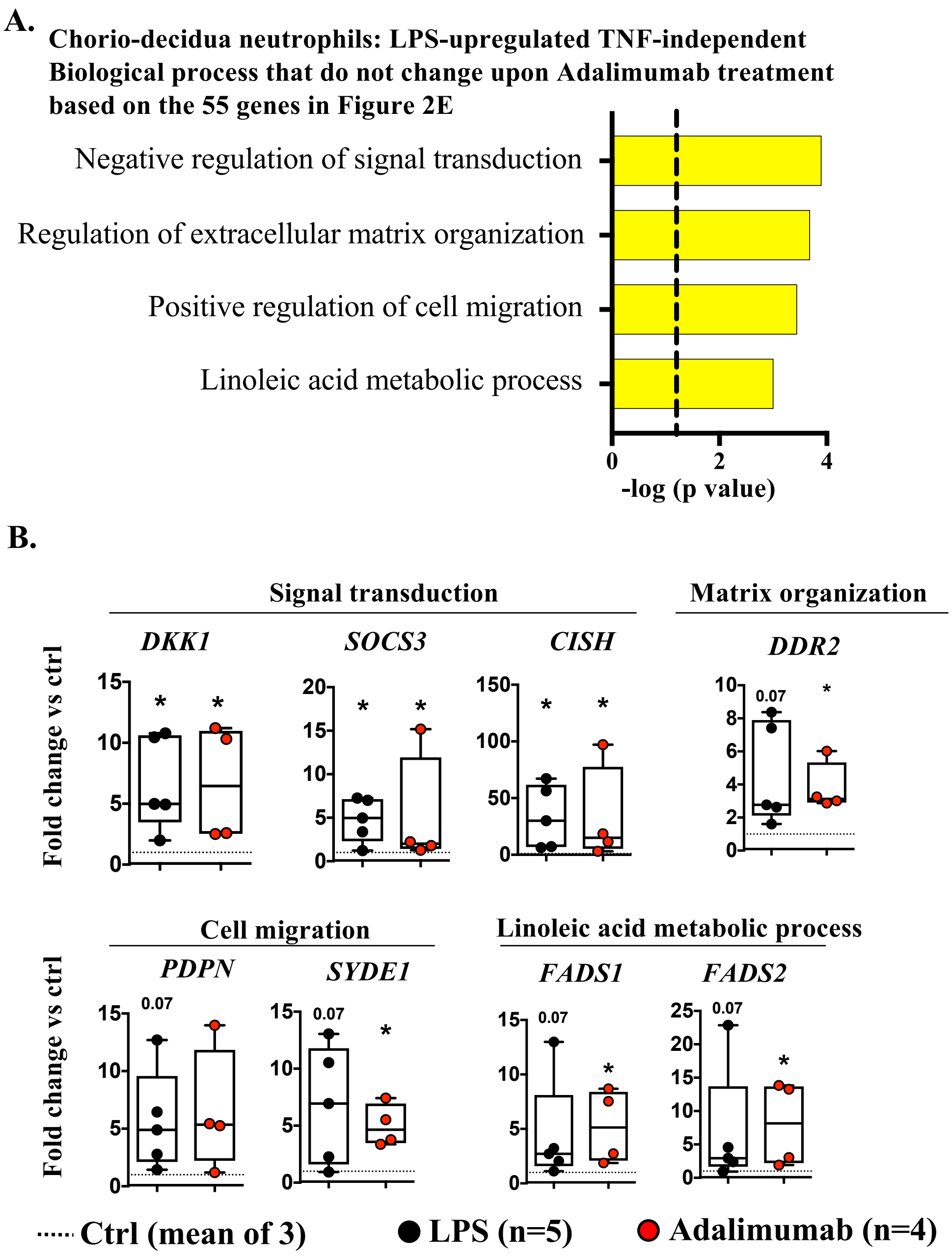

Supplementary Figure 9. LPS regulates signal transduction, extracellular matrix organization, cell migration, linoleic acid metabolic process. **(A)** Biological processes of differentially expressed LPS-upregulated gene, as determined using Enrichr. **(B)** Representative genes associated to the biological processes induced by LPS. (dotted-line represents the mean of 3 Ctrl; LPS n=5; Adalimumab n=4). P * <0.05 vs. ctrl (Mann–Whitney U test).
