# Supplementary material for: TNF-Signaling Modulates Neutrophil-Mediated Immunity at the Feto-Maternal Interface During LPS-Induced Intrauterine Inflammation
Source: Front Immunol. 2020 Apr 3;11:558. doi: 10.3389/fimmu.2020.00558 (PMC7145904; doi:10.3389/fimmu.2020.00558)
Supplement: Supplementary file 11 [file Image_10.pdf]

Supplementary Figure 10.

A.

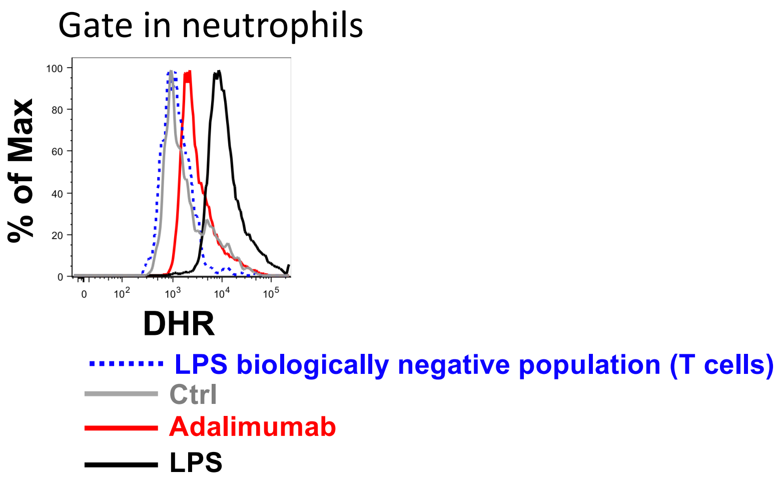

B.

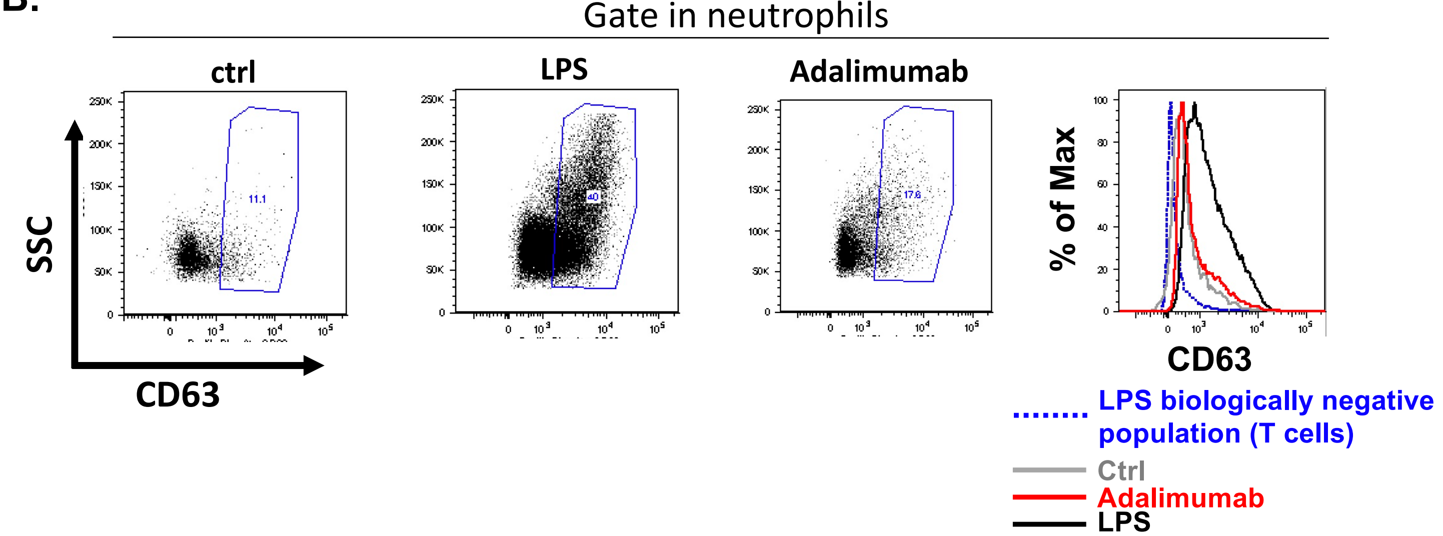

C.

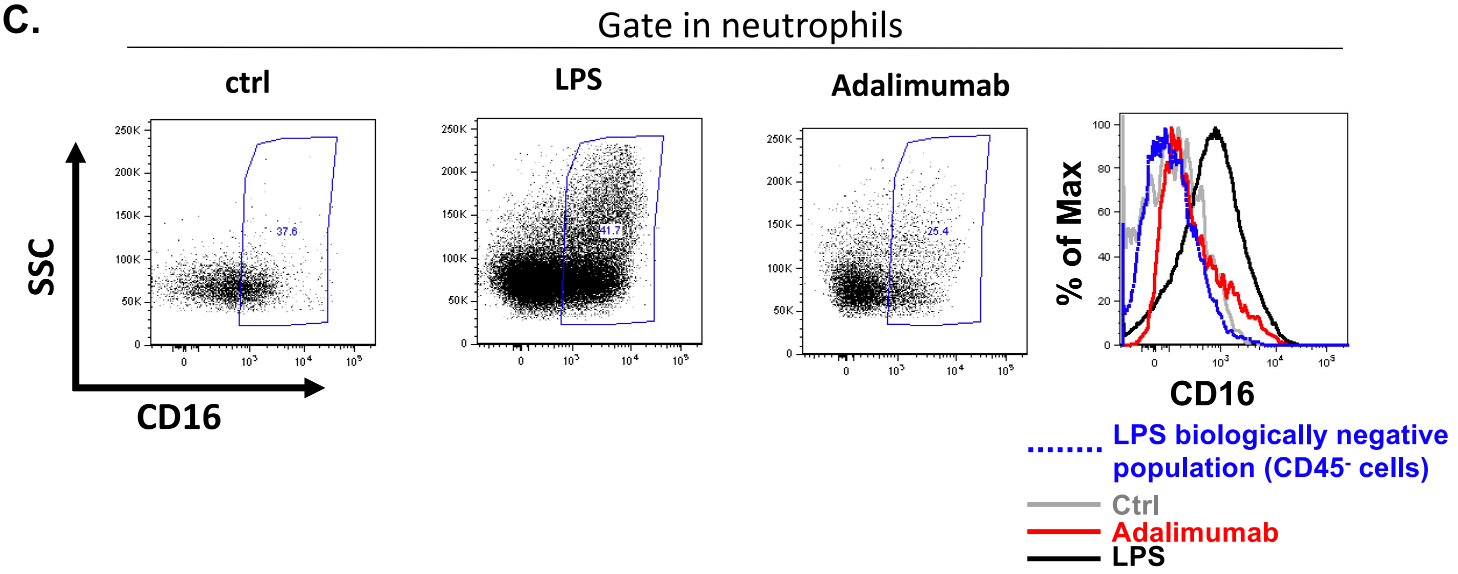

Supplementary Figure 10. Representative dot plots (n=5) showing activation of chorio-decidua Neutrophils. Chorio-decidua cell suspension were analyzed by flow cytometry. Representative mean fluorescence intensity (MFI) of **(A)** Dihydrorhodamine 123 (DHR) - an uncharged and nonfluorescent reactive oxygen species indicator – and frequency and MFI of **(B)** $CD63^{+}$, and **(C)** $CD16^{+}$ neutrophils decreased upon Adalimumab treatment.
