# Supplementary material for: TNF-Signaling Modulates Neutrophil-Mediated Immunity at the Feto-Maternal Interface During LPS-Induced Intrauterine Inflammation
Source: Front Immunol. 2020 Apr 3;11:558. doi: 10.3389/fimmu.2020.00558 (PMC7145904; doi:10.3389/fimmu.2020.00558)
Supplement: Supplementary file 12 [file Image_11.pdf]

Supplementary Figure 11.

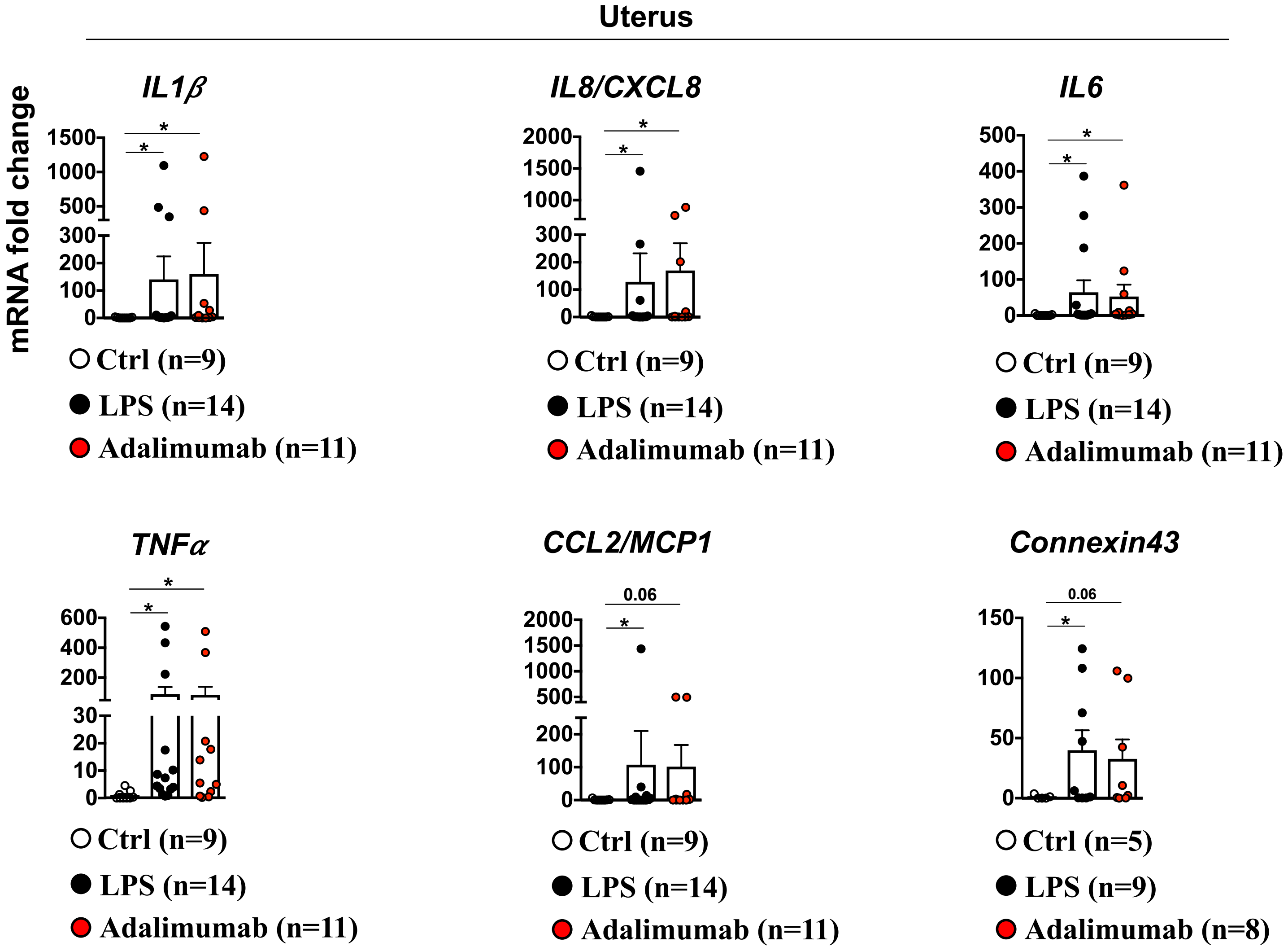

Supplementary Figure 11. Adalimumab did not decrease inflammation in uterus. mRNAs were isolated from full-thickness uterine tissue including the adherent decidua. qPCR was performed using rhesus-specific Taqman probes. The values were first internally normalized to the endogenous 18S RNA, and the resultant values for the experimental animals were shown as fold increase compared with the mean control value (Ctrl n=5-9; LPS n=14; Adalimumab n=8-11). Data are mean ± SEM, *P < .05 vs controls (Mann–Whitney U test).
